# Dynamics of dipole in a stationary non-homogeneous electromagnetic field

**DOI:** 10.1038/s41598-021-96913-4

**Published:** 2021-09-07

**Authors:** Maria Przybylska, Andrzej J. Maciejewski

**Affiliations:** 1 Institute of Physics, University of Zielona Góra, Licealna 9, 65-417 Zielona Góra, Poland; 2 Janusz Gil Institute of Astronomy, University of Zielona Góra, ul. Licealna 9, 65-417 Zielona Góra, Poland

**Keywords:** Mathematics and computing, Physics

## Abstract

The non-relativistic equations of motion for a dipole in a stationary non-homogeneous electromagnetic field are derived and analysed. It is shown that they are Hamiltonian with respect to a certain degenerated Poisson structure. Described by them dynamics is complex because the motion of the centre of mass of the dipole is coupled with its rotational motion. The problem of the existence of linear in momenta first integrals which can be useful for the separation of rotational motion is discussed. The presence of such first integral appears to be related with a linear symmetry of electric and magnetic fields. Also results of search of quadratic in momenta first integrals for uniform and stationary electromagnetic fields are reported. Deriving equations of motion of a dipole in arbitrary stationary electromagnetic fields and analysis of described by them dynamics is important for the construction of electromagnetic traps for polar particles.

## Introduction

The main aim of this paper is to give a general framework for a study dynamics of electric dipole in a stationary non-homogeneous electromagnetic field. In our considerations a dipole is modelled by two equal, and opposite sign point charges. The distance between them is fixed, and the masses of the charges are not necessarily equal. From the dynamical point of view, such a dipole is a degenerated charged rigid body—one of its principal moments of inertia vanishes. Our goal is to derive and investigate equations of motion in the most general case when the mass centre motion of the dipole is coupled with its rotation.

Motivations for this investigation come from our study of electromagnetic traps for charged and neutral particles^1–5^. More specifically, in^5^ we investigated the motion of a small dipole in a special configuration of non-homogeneous electromagnetic field that was proposed as a model of a trap for polar particles. These investigations showed that for a study similar traps we have to construct a general description of a dipole in an electromagnetic field of arbitrary form.

We showed in^4^ that even in a stationary homogeneous electromagnetic field the motion of a dipole is complex and, in general, it is chaotic. If the field is not homogeneous, then having in mind possible physical applications, it makes sense to assume that the dipole is small. But then it is not clear what are the properties of reasonable approximations of the equations. In the commonly used approximation a dipole is just a neutral point mass which carry a dipole moment. However considering polar particles we have to take into account that they have finite dimensions and that their mass distribution in not spherically symmetric. Moreover, the charge and the mass distributions of a polar particle do not need to be necessarily proportional to each other. This is why in our considerations we assume that the dipole is small but we take into account that it can be inertially non-symmetric. That is, its mass centre does not necessarily coincide with its centre of the charge distribution. This asymmetry is described by the parameter $\delta =l(m_2-m_1)/(m_1+m_2)$, where $m_1$ and $m_2$ are the masses of the charges and *l* is the charges separation distance.

The Hamiltonian formalism gives appropriate tools and language for classical description of motion. However, considering problems where electromagnetic is involved the canonical description can be done in two ways, see^4^. For a single charge *q* of mass *m* in an electromagnetic field the canonical momentum conjugated to its position $\user2{r}$ is $\user2{P}=m {{\dot{\user2{r}}}} + q\user2{A}(\user2{r})$, where $\user2{A}(\user2{r})$ is a vector potential so that $\user2{B}(\user2{r})=\nabla \times \user2{A}(\user2{r})$ is the magnetic field. However, it is convenient to use variables $\user2{r}$ and linear momentum $\user2{p}=m{{\dot{\user2{r}}}}$. Of course, in these variables the equations of motion are still Hamiltonian but with respect to a non-canonical symplectic structure which depends on the magnetic field, see, for example^4,6^. The advantage is that the system is gauge invariant. The equations of motion for a dipole can be obtained by a restriction of non-canonical Hamilton’s equations for two charges. The constrain, the constant distance between charges, is holonomic so one can expect that the constrained system is Hamiltonian, and, it is. The other way is just to start from the Lagrangian formulation and use local generalised coordinates. However, the first approach needs not so well known theoretical tools, and the second gives highly complicated formulae.

This is why we choose simple and direct method to derive the equations of motion for a dipole. We just use the Newton–Lorenz equations for a charge in an electromagnetic field and the basic laws of mechanics. We demonstrated it for a dipole in a stationary homogeneous electromagnetic field in^4^. In this article we show that this method works in a general case. The derived equations are Hamiltonian with respect to a degenerated Poisson bracket and they are starting point for our further analysis.

The key goal of out analysis was derivation of equations of motion for a small dipole. To this end we expand into the power series the right hand sides of general equations and truncate them at the first term proportional to the asymmetry parameter. We proved that these equations are Hamiltonian with respect to an appropriate Poisson bracket. This fact is not evident.

As we already mentioned the motion of the centre of mass of the dipole is coupled with its rotational motion. The centre of mass does not move with constant velocity. Nevertheless, if the electromagnetic field is stationary and homogeneous, then existence of first integrals linear in the momenta allows to separate equations for the rotational motion of the dipole, see^4^ for details. This is why it is important to know whether the obtained equations of motion of the dipole admit first integrals linear in momenta. We analyse this problem in details and show among other things that if such a first integral exits, then the vector fields $\user2{E}(\user2{r})$ and $\user2{B}(\user2{r})$ have a certain linear symmetry field $\user2{S}(\user2{r})$, that is they both commute with $\user2{S}(\user2{r})$: $[\user2{E}(\user2{r}),\user2{S}(\user2{r})]=[\user2{B}(\user2{r}),\user2{S}(\user2{r})]=\user2{0}$, where $[\cdot ,\cdot ]$ means the Lie bracket of vector fields.

The plan of this paper is as follows. In Section “Equations of motion” we derive equations of motion of an electric dipole in arbitrary time-independent electromagnetic fields using the Newton-Lorentz equations of translational and rotational motion. Motion of the dipole is governed by four vector equations which describe the motion of its centre of mass and rotations of the dipole depending on values of electric and magnetic fields at positions of two charges creating the dipole. These equations are Hamiltonian with respect to a certain degenerated Poisson structure with two Casimir functions. In Section “Equations of motion of a small dipole”, we derive truncated equations of motion of the dipole under assumptions that its dimension is small with respect to the size of non-linearities of the electromagnetic field and in expansions terms up to the second order with respect to length of the dipole are preserved. It is interesting that these equations are also Hamiltonian with the Poisson structure that is the truncation of the Poisson structure from the previous section and that has the same two Casimir functions. Section “First integrals” contains results of the research of first integrals using the direct method. The most general results are obtained for the case when searched first integral is linear in linear momentum of the centre of mass of the dipole, coordinates of the dipole vector and angular velocity of rotational motion of the dipole. The forms of such first integrals and expressions for the electromagnetic field admitting such first integrals are given. For first integrals quadratic in the mentioned variables we were able to finish analysis only for stationary homogeneous electromagnetic fields and these results are given in Section “Final notes and comments”. Also in this section we discuss limits of applicability of the presented model of an electric dipole. In Appendices 1 and 2, Lagrange equations for the cases of the dipole in electromagnetic fields without the smallness of the dipole assumption and with this assumption are given, respectively. We underline that in our considerations we do not assume that masses of two opposite point charges are the same as it is usually made.

## Equations of motion

In this section, we derive equations of non-relativistic motion of an electric dipole in a time independent electromagnetic field without assumption about the smallness of the dipole with respect to the size of non-linearities of the electromagnetic field. We show that it is possible to present them as Hamilton’s equations with respect to a certain degenerated Poisson structure.

At first let us fix notation. A vector $\user2{x}\in \mathbb {R}^n$ will be considered as a one column matrix $\user2{x}=\left[ x_1, \ldots , x_n \right] ^T$. Thus, the scalar product $\user2{x}\cdot \user2{y}$ of two vectors is $\user2{x}\cdot \user2{y}=\user2{x}^T\user2{y}=\user2{y}^T\user2{x}$. The gradient $\nabla f(\user2{x})$ of a scalar function $f(\user2{x})$ is considered as a vector, and we denote $f'(\user2{x}):=\mathrm {d}f(\user2{x}):=(\nabla f(\user2{x}))^T$. With this convention $f'(\user2{x})\user2{a}=\user2{a}\cdot \nabla f(\user2{x})$ for a vector $\user2{a}\in \mathbb {R}^n$. For a vector function

1 $$\begin{aligned} \mathbb {R}^n \ni \user2{x}\mapsto \user2{F}(\user2{x})= \left[ F_1(\user2{x}), \ldots , F_m(\user2{x})\right] ^T\in \mathbb {R}^m, \end{aligned}$$

we denote

2 $$\begin{aligned} \user2{F}'(\user2{x})= \begin{bmatrix} F_1'(\user2{x})\\ \vdots \\ F_m'(\user2{x}) \end{bmatrix} = \left[ \nabla F_1(\user2{x}), \ldots , \nabla F_m(\user2{x})\right] ^T \end{aligned}$$

and

3 $$\begin{aligned} \user2{F}'(\user2{x})\user2{a}=\begin{bmatrix} F_1'(\user2{x})\user2{a}\\ \vdots \\ F_m'(\user2{x})\user2{a}\end{bmatrix}, \quad \user2{a}\cdot \user2{F}'(\user2{x})=\user2{a}^T\user2{F}'(\user2{x})= \sum _{i=1}^n a_i F_i'(\user2{x}), \quad \quad \text {for}\quad \user2{a}\in \mathbb {R}^n. \end{aligned}$$

The Hessian $f''(\user2{x})$ of a scalar function $f(\user2{x})$ is

4 $$ \begin{gathered}   f^{{\prime \prime }} (x): = \left[ {\begin{array}{*{20}c}    {\frac{{\partial ^{2} f}}{{\partial x_{1} ^{2} }}(x),\quad  \ldots \quad \frac{{\partial ^{2} f}}{{\partial x_{n} \partial x_{1} }}(x)}  \\    { \ldots  \ldots  \ldots  \ldots  \ldots  \ldots  \ldots }  \\    {\frac{{\partial ^{2} f}}{{\partial x_{1} \partial x_{n} }}(x),\; \ldots \quad \frac{{\partial ^{2} f}}{{\partial x_{n} ^{2} }}(x)}  \\   \end{array} } \right] \hfill \\    \hfill \\  \end{gathered}  $$

and we denote $f''(\user2{x})(\user2{a},\user2{b}):=\user2{a}^Tf''(\user2{x})\user2{b}$, where $\user2{a},\user2{b}\in \mathbb {R}^n$. For a vector function (1) we set

5 $$\begin{aligned} \user2{F}''(\user2{x}):= \begin{bmatrix} F_1''(\user2{x})\\ \vdots \\ F_m''(\user2{x}) \end{bmatrix}, \quad \user2{F}''(\user2{x})(\user2{a},\user2{b}):= \begin{bmatrix} \user2{a}^TF_1''(\user2{x})\user2{b}\\ \vdots \\ \user2{a}^TF_m''(\user2{x})\user2{b}\end{bmatrix}\quad \text {and}\quad \user2{a}\cdot \user2{F}''(\user2{x})= \sum _{i=1}^n a_i F_i''(\user2{x}). \end{aligned}$$

For ${\user2{x}}, {\user2{y}}\in \mathbb {R}^3$ the scalar and vector products we denote by ${\user2{x}}\cdot {\user2{y}}$ and ${\user2{x}}\times {\user2{y}}$, respectively. For a vector ${\user2{x}}\in \mathbb {R}^3$, the corresponding skew-symmetric matrix is

6 $$\begin{aligned} {{\widehat{{\user2{x}}}}} := \begin{bmatrix} 0 &{} -x_3 &{} x_2 \\ x_3 &{} 0 &{} -x_1 \\ -x_2 &{} x_1 &{} 0 \end{bmatrix}. \end{aligned}$$

The map ${\user2{x}}\mapsto {\widehat{{\user2{x}}}}$ is the standard isomorphism between Lie algebras $\mathfrak {so}(3,\mathbb {R})$ and $\mathbb {R}^3$ with the vector product as algebra multiplication. It has the following properties

7 $$\begin{aligned} {{\widehat{{\user2{x}}}}}{\user2{y}}={\user2{x}}\times {\user2{y}}, \qquad \widehat{{\user2{x}}\times {\user2{y}}}={{\widehat{{\user2{x}}}}}{{\widehat{{\user2{y}}}}}- {{\widehat{{\user2{y}}}}}{{\widehat{{\user2{x}}}}}={\user2{y}}^T{\user2{x}}-{\user2{x}}^T{\user2{y}}, \qquad {{\widehat{{\user2{x}}}}}{{\widehat{{\user2{y}}}}} = {\user2{y}}{\user2{x}}^T- ({\user2{x}}^T{\user2{y}}){\mathrm {Id}}_3, \end{aligned}$$

where ${\mathrm {Id}}_3$ is the three-dimensional identity matrix. Moreover, for an arbitrary $3\times 3$ matrix ${\user2{M}}\in \mathbb {M}(3,\mathbb {R})$ the following identity

8 $$\begin{aligned} {\widehat{{\user2{x}}}}{\user2{M}}+{\user2{M}}^T{\widehat{{\user2{x}}}} + \widehat{{\user2{M}}{\user2{x}}}= ({\text {Tr}}{\user2{M}}){\widehat{{\user2{x}}}} \end{aligned}$$

holds true.

Let us recall that motion of a point with mass *m*, electric charge *q* and radius vector ${\user2{r}}$ in a stationary electromagnetic field is described by the Newton–Lorentz equation

9 $$\begin{aligned} m{\ddot{{\user2{r}}}} = q \left[ {\user2{E}}({\user2{r}}) + {{\dot{{\user2{r}}}}} \times {\user2{B}}({\user2{r}}) \right] , \end{aligned}$$

where ${\user2{E}}({\user2{r}})$ and ${\user2{B}}({\user2{r}})$ denote the electrostatic and magnetostatic fields, respectively.

**Figure 1:**
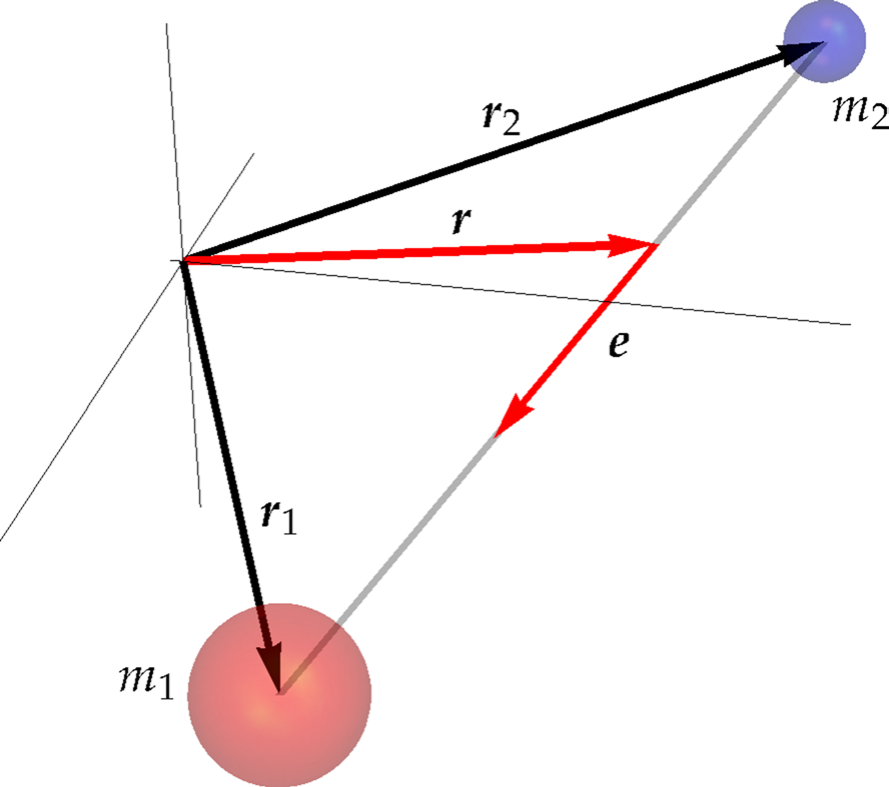
Model of a dipole.

We consider a dipole which is modelled by two charges $+q$ and $-q$ with the respective masses $m_1$ and $m_2$. Their positions in an inertial frame are given by radius vectors ${\user2{r}}_1$ and ${\user2{r}}_2$, see Fig. 1. The centre of mass of the dipole is localised at

10 $$\begin{aligned} {\user2{r}}=\frac{1}{m}(m_1{\user2{r}}_1+m_2{\user2{r}}_2), \qquad m=m_1+m_2, \end{aligned}$$

hence

11 $$\begin{aligned} {\user2{r}}_1={\user2{r}}+\frac{m_2}{m}l {\user2{e}},\quad {\user2{r}}_2={\user2{r}}-\frac{m_1}{m}l{\user2{e}}, \end{aligned}$$

or

12 $$\begin{aligned} {\user2{r}}_1={\user2{r}}+\frac{1}{2}(\delta +l){\user2{e}},\qquad {\user2{r}}_1= {\user2{r}}+\frac{1}{2}(\delta -l){\user2{e}},\qquad \delta =\frac{m_2-m_1}{m}l. \end{aligned}$$

Here ${\user2{r}}_1-{\user2{r}}_2= l {\user2{e}}$, where ${\user2{e}}$ is the unity vector $l=|{\user2{r}}_1-{\user2{r}}_2|$ is the distance between charges. Thus, the configuration space of the dipole is $\mathbb {R}^3\times {\mathbb {S}}^2$, where ${\mathbb {S}}^2\subset \mathbb {R}^3$ is the unite sphere. The force acting on the centre of mass of the dipole reads

13 $$\begin{aligned} {\user2{F}}=q\left[ {\user2{E}}({\user2{r}}_1)+{\dot{{\user2{r}}}}_1\times {\user2{B}}({\user2{r}}_1)\right] - q\left[ {\user2{E}}({\user2{r}}_2)+{\dot{{\user2{r}}}}_2\times {\user2{B}}({\user2{r}}_2)\right] , \end{aligned}$$

so its motion is described by Newton’s equation

14 $$\begin{aligned} m{\ddot{{\user2{r}}}} = q\left[ {\user2{E}}({\user2{r}}_1)+{\dot{{\user2{r}}}}_1\times {\user2{B}}({\user2{r}}_1)\right] - q\left[ {\user2{E}}({\user2{r}}_2)+{\dot{{\user2{r}}}}_2\times {\user2{B}}({\user2{r}}_2)\right] . \end{aligned}$$

Notice that the right hand side of this equation depends only on $({\user2{r}},{\user2{e}})$ and $({{\dot{{\user2{r}}}}},{{\dot{{\user2{e}}}}})$.

The total angular momentum of the dipole is

15 $$\begin{aligned} {\user2{L}}=m_1{\user2{r}}_1\times {\dot{{\user2{r}}}}_1 + m_2{\user2{r}}_2\times {\dot{{\user2{r}}}}_2 =m {\user2{r}}\times {\dot{{\user2{r}}}}+\frac{m_1m_2}{m}l^2\, {\user2{e}}\times {\dot{{\user2{e}}}}= m {\user2{r}}\times {\dot{{\user2{r}}}}+I\varvec{\omega }, \end{aligned}$$

where we denoted

16 $$\begin{aligned} I=\frac{m_1m_2}{m}l^2, \qquad \varvec{\omega }={\user2{e}}\times {\dot{{\user2{e}}}}. \end{aligned}$$

Thus ${\user2{e}}\cdot \varvec{\omega }=0$, so we can write ${{\dot{{\user2{e}}}}}= \varvec{\omega }\times {\user2{e}}$.

The torque of electrostatic forces acting on the dipole is

17 $$\begin{aligned} {\user2{K}}=q{\user2{r}}_1\times \left[ {\user2{E}}({\user2{r}}_1)+{\dot{{\user2{r}}}}_1\times {\user2{B}}({\user2{r}}_1)\right] -q{\user2{r}}_2\times \left[ {\user2{E}}({\user2{r}}_2)+{\dot{{\user2{r}}}}_2\times {\user2{B}}({\user2{r}}_2)\right] . \end{aligned}$$

Taking into account identities (12) the Newton equation (14) can be written in the form

18 $$\begin{aligned} m{\ddot{{\user2{r}}}}=q\left[ {\user2{E}}({\user2{r}}_1)-{\user2{E}}({\user2{r}}_2)\right] +q{{\dot{{\user2{r}}}}}\times \left[ {\user2{B}}({\user2{r}}_1)-{\user2{B}}({\user2{r}}_2)\right] +\frac{q}{2}{{\dot{{\user2{e}}}}}\times \Big [(\delta +l){\user2{B}}({\user2{r}}_1)-(\delta -l){\user2{B}}({\user2{r}}_2) \Big ], \end{aligned}$$

and for the torque (17) takes the form

19 $$\begin{aligned} \begin{aligned} {\user2{K}}&=q{\user2{r}}\times \left[ {\user2{E}}({\user2{r}}_1)-{\user2{E}}({\user2{r}}_2)\right] +q{\user2{r}}\times \left[ {{\dot{{\user2{r}}}}}\times {\user2{B}}({\user2{r}}_1)- {{\dot{{\user2{r}}}}}\times {\user2{B}}({\user2{r}}_2)\right] \\&\quad + \frac{q}{2}{\user2{r}}\times \Big [(\delta +l){{\dot{{\user2{e}}}}}\times {\user2{B}}({\user2{r}}_1)- (\delta -l){{\dot{{\user2{e}}}}}\times {\user2{B}}({\user2{r}}_2)\Big ]\\&\quad + \frac{q}{2}{\user2{e}}\times \Big [(\delta +l){\user2{E}}({\user2{r}}_1) -(\delta -l){\user2{E}}({\user2{r}}_2)\Big ]+ \frac{q}{2}{\user2{e}}\times \Big [(\delta +l){{\dot{{\user2{r}}}}}\times {\user2{B}}({\user2{r}}_1)- (\delta -l){{\dot{{\user2{r}}}}}\times {\user2{B}}({\user2{r}}_2)\Big ]\\&\quad + \frac{q}{4}{\user2{e}}\times \Big [(\delta +l)^2{{\dot{{\user2{e}}}}}\times {\user2{B}}({\user2{r}}_1)- (\delta -l)^2{{\dot{{\user2{e}}}}}\times {\user2{B}}({\user2{r}}_2)\Big ]. \end{aligned} \end{aligned}$$

The time derivation of ${\user2{L}}$, see (15), is

20 $$\begin{aligned} {{\dot{{\user2{L}}}}}=m{\user2{r}}\times \ddot{{\user2{r}}}+I{\dot{\varvec{\omega }}}. \end{aligned}$$

As the Newton equation for the rotational motion reads

21 $$\begin{aligned} {\frac{\mathrm {d}}{\mathrm {d}t}}{\user2{L}}= {\user2{K}}, \end{aligned}$$

taking into account equalities (18) and (20), after some simplifications, we obtain

22 $$\begin{aligned} \begin{aligned} I{{\dot{\varvec{\omega }}}}&= \frac{q}{2}{\user2{e}}\times \Big [(\delta +l){\user2{E}}({\user2{r}}_1) -(\delta -l){\user2{E}}({\user2{r}}_2)\Big ] + \frac{q}{2}{\user2{e}}\times \left( {{\dot{{\user2{r}}}}}\times \Big [(\delta +l){\user2{B}}({\user2{r}}_1) -(\delta -l){\user2{B}}({\user2{r}}_2)\Big ]\right) \\&\quad + \frac{q}{4}\Big [(\delta +l)^2{\user2{e}}\cdot {\user2{B}}({\user2{r}}_1)- (\delta -l)^2{\user2{e}}\cdot {\user2{B}}({\user2{r}}_2)\Big ]{{\dot{{\user2{e}}}}} \end{aligned} \end{aligned}$$

Let us define the linear momentum ${\user2{p}}=m{{\dot{{\user2{r}}}}}$ and the rotational angular momentum ${\user2{g}}=I\omega =I{\user2{e}}\times {{\dot{{\user2{e}}}}}$. Then Eqs. (18) and (22) can be rewritten as the following system of the first order equations

23 $$\begin{aligned} \begin{aligned} {{\dot{{\user2{r}}}}}&=\frac{1}{m}{\user2{p}},\\ {{\dot{{\user2{p}}}}}&=q\left[ {\user2{E}}({\user2{r}}_1)-{\user2{E}}({\user2{r}}_2)\right] +\frac{q}{m}{\user2{p}}\times \left[ {\user2{B}}({\user2{r}}_1)-{\user2{B}}({\user2{r}}_2)\right] +\frac{q}{2I}({\user2{g}}\times {\user2{e}})\times \Big [ (\delta +l){\user2{B}}({\user2{r}}_1) -(\delta -l){\user2{B}}({\user2{r}}_2) \Big ],\\ {{\dot{{\user2{e}}}}}&=\frac{1}{I}{\user2{g}}\times {\user2{e}},\\ {{\dot{{\user2{g}}}}}&=\frac{q}{2}{\user2{e}}\times \left[ (\delta +l){\user2{E}}({\user2{r}}_1)- (\delta -l){\user2{E}}({\user2{r}}_2)\right] +\frac{q}{2m}{\user2{e}}\times \Big [{\user2{p}}\times \Big ((\delta +l) {\user2{B}}({\user2{r}}_1) -(\delta -l) {\user2{B}}({\user2{r}}_2)\Big )\Big ]\\&\quad +\frac{q}{4I}\Big [ (\delta +l)^2{\user2{e}}\cdot {\user2{B}}({\user2{r}}_1)-(\delta -l)^2{\user2{e}}\cdot {\user2{B}}({\user2{r}}_2) \Big ]({\user2{g}}\times {\user2{e}}). \end{aligned} \end{aligned}$$

In Appendix 1, we show that this system can be derived from appropriate Lagrange equations.

System (23) has the energy integral

24 $$\begin{aligned} H = \frac{1}{2m} {\user2{p}}\cdot {\user2{p}}+ \frac{1}{2I}{\user2{g}}\cdot {\user2{g}}+q\Phi ({\user2{r}}_1)-q\Phi ({\user2{r}}_2), \end{aligned}$$

where $\Phi ({\user2{r}})$ is the scalar potential of the electromagnetic field, that is ${\user2{E}}({\user2{r}})=-\nabla \Phi ({\user2{r}})$. Moreover, it has two additional first integrals

25 $$\begin{aligned} H_0={\user2{e}}\cdot {\user2{e}}, \qquad H_1={\user2{e}}\cdot {\user2{g}}. \end{aligned}$$

Let us denote ${\user2{x}}=({\user2{r}},{\user2{p}},{\user2{e}},{\user2{g}})\in \mathbb {R}^{12}$ and define the following $12\times 12$ matrix

26 $$\begin{aligned} \varvec{\Pi }({\user2{x}}):= \begin{bmatrix} \varvec{0}&{} {\mathrm {Id}}_3 &{} \varvec{0}&{} \varvec{0}\\ -{\mathrm {Id}}_3 &{}{\widehat{{\user2{b}}}} &{} \varvec{0}&{} \varvec{\Omega }\\ \varvec{0}&{} \varvec{0}&{}\varvec{0}&{} -{{\widehat{{\user2{e}}}}} \\ \varvec{0}&{} -\varvec{\Omega }^T &{} -{\widehat{{\user2{e}}}} &{} {{\widehat{\varvec{\Xi }}}} \end{bmatrix}, \end{aligned}$$

where

27 $$\begin{aligned} \begin{aligned} {\user2{b}}&= -q{{\user2{B}}({\user2{r}}_1)}+q{{\user2{B}}({\user2{r}}_2)},\qquad \varvec{\Omega }=\frac{q}{2}{{\widehat{{\user2{e}}}}} \left[ (\delta +l)\widehat{{\user2{B}}({\user2{r}}_1)} - (\delta -l) \widehat{{\user2{B}}({\user2{r}}_2)} \right] ,\\ \varvec{\Xi }&=-\frac{q}{4}\left[ (\delta +l)^2{\user2{e}}\cdot {\user2{B}}({\user2{r}}_1)- (\delta -l)^2{\user2{e}}\cdot {\user2{B}}({\user2{r}}_2)\right] {\user2{e}}-{\user2{g}}. \end{aligned} \end{aligned}$$

Then we can write system (23) in the form

28 $$\begin{aligned} {\frac{\mathrm {d}}{\mathrm {d}t}}{\user2{x}}= \varvec{\Pi }({\user2{x}}) H'({\user2{x}}). \end{aligned}$$

Moreover using matrix $\varvec{\Pi }({\user2{x}})$ we can define the following bracket

29 $$\begin{aligned} \{F,G\}({\user2{x}}):=F'({\user2{x}})^T\varvec{\Pi }({\user2{x}})G'({\user2{x}}), \end{aligned}$$

where $F({\user2{z}})$ and $G({\user2{z}})$ are smooth functions.

### Lemma 2.1

*Bracket defined by* (29) *is a Poisson bracket by Gauss’s law for magnetism* $\nabla \cdot {\user2{B}}({\user2{r}})=0$. *Moreover* $H_0$ *and* $H_1$ *are its Casimir functions.*

### Proof

Facts that the bracket is bilinear, antisymmetric and satisfies the Leibniz identity follow directly from its definition. It is also simply to show that $\varvec{\Pi }({\user2{x}})H_0'({\user2{x}})=\varvec{0}$ and $\varvec{\Pi }({\user2{x}})H_1'({\user2{x}})=\varvec{0}$. However checking the Jacobi identity is not trivial. Let us denote expressions

30 $$\begin{aligned} J_{i,j,k}:=\{x_i,\{x_j,x_k\}\}+\{x_k,\{x_i,x_j\}\}+\{x_j,\{x_k,x_i\}\}, \end{aligned}$$

for $$i,j,k\in \{1,\ldots ,12\}$$. Taking into account the antisymmetry of the bracket it is enough to show that $$ J_{i,j,k}=0$$ for $$1\le i<j<k\le 12$$. From the form of matrix $$\varvec{\Pi }({\user2{x}})$$ it is easy to deduce that $$\{r_i, x_j \}=\delta _{j,3+i}$$ for $$i=1,2,3$$ and $$j\in \{1,\ldots ,12\}$$. Thus, $$J_{i,j,k}=0$$ for $$i<4$$. Moreover, $$\{e_i,x_j\}=0$$ for $$j<10$$, and $$\{e_i,g_j\}=\varepsilon _{i,j,k}e_k$$, so $$J_{i,j,k}=0$$ for $$ i=7,8,9$$.

Direct calculations that for remaining values of indices the are 16 cases for which $$ J_{i,j,k}\ne 0$$, however for these cases $$ J_{i,j,k}$$ is a linear homogeneous polynomial of $$ \nabla \cdot {\user2{B}}({\user2{r}}_1) $$ and $$ \nabla \cdot {\user2{B}}({\user2{r}}_2) $$. For example one can obtain

31 $$\begin{aligned} \begin{aligned} J_{4,5,6}&=q \left[ \nabla \cdot {\user2{B}}({\user2{r}}_2) - \nabla \cdot {\user2{B}}({\user2{r}}_1) \right] ,\\ J_{4,5,10}&=-\frac{1}{2}qe_{2} \left[ (1-\delta )\nabla \cdot {\user2{B}}({\user2{r}}_2) +(1+\delta ) \nabla \cdot {\user2{B}}({\user2{r}}_1) \right] ,\\ J_{4,10,11}&=\frac{1}{4}qe_1 e_{3}\left[ (1-\delta )^2\nabla \cdot {\user2{B}}({\user2{r}}_2) -(1+\delta )^2 \nabla \cdot {\user2{B}}({\user2{r}}_1) \right] .\\ \end{aligned} \end{aligned}$$

Hence, if $$\nabla \cdot {\user2{B}}({\user2{r}}) =0$$, then $$ J_{i,j,k}=0$$ for $$1\le i<j<k\le 12$$, and this finishes our proof. $$\square $$

## Equations of motion of a small dipole

If the dimension of the dipole is small with respect to the size of non-linearities of the fields, then we can obtain approximate equations of motion just expanding the force and the torque acting on the dipole into power series and truncate these expansions at appropriate order. In our considerations we need expansions up to the second order with respect to *l*. For calculations it is convenient to introduce the following notation

32 $$\begin{aligned} \varvec{\Delta }_1= \frac{m_2}{m}l {\user2{e}}\qquad \varvec{\Delta }_2=-\frac{m_1}{m}l{\user2{e}}\qquad \delta =\frac{m_2-m_1}{m}l. \end{aligned}$$

Then

33 $$\begin{aligned} \varvec{\Delta }_1- \varvec{\Delta }_2= l{\user2{e}}, \qquad \varvec{\Delta }_1+ \varvec{\Delta }_2= \delta {\user2{e}}, \end{aligned}$$

and so we get

34 $$\begin{aligned} \varvec{\Delta }_1= \frac{1}{2}(\delta +l){\user2{e}}, \qquad \varvec{\Delta }_2= \frac{1}{2}(\delta -l){\user2{e}}. \end{aligned}$$

Now, for an arbitrary $3\times 3$ matrix ${\user2{M}}$ we have

35 $$\begin{aligned} \varvec{\Delta }_1\cdot {\user2{M}}\varvec{\Delta }_1 - \varvec{\Delta }_2\cdot {\user2{M}}\varvec{\Delta }_2 = l\delta {\user2{e}}\cdot {\user2{M}}{\user2{e}}\end{aligned}$$

and

36 $$\begin{aligned} {{\dot{\varvec{\Delta }}}}_1\times {\user2{M}}\varvec{\Delta }_1 - {{\dot{\varvec{\Delta }}}}_2\times {\user2{M}}\varvec{\Delta }_2 = \delta l{{\dot{{\user2{e}}}}}\times {\user2{M}}{\user2{e}}. \end{aligned}$$

With the above identities we obtain the following expansions

37 $$\begin{aligned} {\user2{E}}({\user2{r}}+ \varvec{\Delta }_1 ) -{\user2{E}}({\user2{r}}+ \varvec{\Delta }_2 ) = l{\user2{E}}'({\user2{r}}){\user2{e}}+ \frac{1}{2}\delta l {\user2{E}}''({\user2{r}})({\user2{e}},{\user2{e}}) +{{\mathcal {O}}}(l^3) \end{aligned}$$

and

38 $$\begin{aligned}{}& ({\dot{\user2{r}}}+{{\dot{\varvec{\Delta }}}}_1)\times \user2{B}(\user2{r}+ \varvec{\Delta }_1)-({\dot{\user2{r}}} +{{\dot{\varvec{\Delta }}}}_2)\times \user2{B}(\user2{r}+ \varvec{\Delta }_2) \\& \quad = l {{\dot{\user2{r}}}} \times \user2{B}'(\user2{r})\user2{e}+ l {{\dot{\user2{e}}}} \times \user2{B}(\user2{r}) +\delta l {{\dot{\user2{e}}}}\times \user2{B}'(\user2{r})\user2{e}+ \frac{1}{2}\delta l {{\dot{\user2{r}}}}\times \left( \user2{B}''(\user2{r})(\user2{e},\user2{e})\right) +{{\mathcal {O}}}(l^3). \end{aligned}$$

Hence, the truncated Newton equation of motion $m{\ddot{{\user2{r}}}}={\user2{F}}$ describing the motion of the centre of mass of the dipole has the form

39 $$\begin{aligned} \begin{aligned} m{\ddot{{\user2{r}}}}&= ql \left[ {\user2{E}}'({\user2{r}}){\user2{e}}+{{\dot{{\user2{r}}}}} \times {\user2{B}}'({\user2{r}}){\user2{e}}+ {{\dot{{\user2{e}}}}} \times {\user2{B}}({\user2{r}}) \right] \\&\quad +\frac{1}{2}ql\delta \left[ {\user2{E}}''({\user2{r}})({\user2{e}},{\user2{e}}) + {{\dot{{\user2{r}}}}}\times \left( {\user2{B}}''({\user2{r}})({\user2{e}},{\user2{e}})\right) + 2{{\dot{{\user2{e}}}}}\times {\user2{B}}'({\user2{r}}){\user2{e}}\right] . \end{aligned} \end{aligned}$$

In an electric field this force is non-zero only in an inhomogeneous electric field and we recognise famous expression for this force $ql{\user2{E}}'({\user2{r}}){\user2{e}}=\left( {\user2{d}}\cdot \nabla \right) {\user2{E}}({\user2{r}})$, see, for example, Section 4.1.3 in^7^. Next two terms are related with the action of a magnetic field on electric dipole and are not such frequent in literature but one can find them for example in^8^, see also additional explanations in Section 2.1 of^9^. Expressions for forces in the first row (39) agree with these in Eq. (33) in^8^ remembering that moving electric dipole in a magnetic field also has magnetic moment $\varvec{\mu }={\user2{d}}\times {{\dot{{\user2{r}}}}}$. Expressions for forces given in the second line are present only for asymmetric dipoles with $m_2\ne m_1$ and according to our knowledge are new.

The total angular momentum of the dipole is given in (15) and the expansion of expression (17) for the torque to the second order with respect to *l* gives

40 $$\begin{aligned} \begin{aligned} {\user2{K}}&= d \Big ({\user2{r}}\times \left[ {\user2{E}}'({\user2{r}}){\user2{e}}+{{\dot{{\user2{r}}}}}\times {\user2{B}}'({\user2{r}}){\user2{e}}+{{\dot{{\user2{e}}}}}\times {\user2{B}}({\user2{r}})\right] +{\user2{e}}\times \left[ {\user2{E}}({\user2{r}}) +{{\dot{{\user2{r}}}}}\times {\user2{B}}({\user2{r}})\right] \Big )\\&\quad +\frac{d\delta }{2}\Big ( {\user2{r}}\times \left[ {\user2{E}}''({\user2{r}})({\user2{e}},{\user2{e}})+ {{\dot{{\user2{r}}}}}\times {\user2{B}}''({\user2{r}})({\user2{e}},{\user2{e}}) \right. \\&\quad \left. +2{{\dot{{\user2{e}}}}}\times {\user2{B}}'({\user2{r}}){\user2{e}}\right] +2{\user2{e}}\times \left[ {\user2{E}}'({\user2{r}}){\user2{e}}+{{\dot{{\user2{r}}}}}\times {\user2{B}}'({\user2{r}}){\user2{e}}+{{\dot{{\user2{e}}}}}\times {\user2{B}}({\user2{r}})\right] \Big ). \end{aligned} \end{aligned}$$

Here we also recognise two well known terms of torque for a dipole in an electric field $d{\user2{e}}\times {\user2{E}}({\user2{r}})={\user2{d}}\times {\user2{E}}({\user2{r}})$ and in a non-homogeneous field $d{\user2{r}}\times {\user2{E}}'({\user2{r}}){\user2{e}}={\user2{r}}\times \left( {\user2{d}}\cdot \nabla \right) {\user2{E}}({\user2{r}})$, see, for example, Section 4.1.3 in^7^, less known terms related to the presence of magnetic field, see^8^ and new terms related to the asymmetry of the dipole given in the second and in the third lines of Eq. (40).

Taking into account equalities (39) and (20) the truncated Newton equation for the rotational motion (21) reads

41 $$\begin{aligned} I{{\dot{\varvec{\omega }}}} = d{\user2{e}}\times \left[ {\user2{E}}({\user2{r}})+{\dot{{\user2{r}}}}\times {\user2{B}}({\user2{r}})\right] + d\delta {\user2{e}}\times \left[ {\dot{{\user2{e}}}}\times {\user2{B}}({\user2{r}})+ {\user2{E}}'({\user2{r}}){\user2{e}}+{{\dot{{\user2{r}}}}}\times {\user2{B}}'({\user2{e}}){\user2{e}}\right] . \end{aligned}$$

Using definitions of the linear ${\user2{p}}=m{{\dot{{\user2{r}}}}}$ and the angular momentum ${\user2{g}}=I\varvec{\omega }$ of the dipole, the Newton equations can be rewritten as the system of first order differential equations

42 $$\begin{aligned} \begin{aligned} {{\dot{{\user2{r}}}}}&= \frac{1}{m} {\user2{p}},\\ {{\dot{{\user2{p}}}}}&= d\left[ \frac{1}{I}({\user2{g}}\times {\user2{e}})\times {\user2{B}}+ {\user2{E}}'({\user2{r}}){\user2{e}}+\frac{1}{m}{\user2{p}}\times \left( {\user2{B}}'({\user2{r}}){\user2{e}}\right) \right] \\&\quad +\frac{d\delta }{2}\left[ {\user2{E}}''({\user2{r}})({\user2{e}},{\user2{e}})+\frac{1}{m}{\user2{p}}\times \left( {\user2{B}}''({\user2{r}})({\user2{e}},{\user2{e}})\right) +\frac{2}{I}\left( {\user2{g}}\times {\user2{e}}\right) \times {\user2{B}}'({\user2{r}}){\user2{e}}\right] , \\ {{\dot{{\user2{e}}}}}&= \frac{1}{I}{\user2{g}}\times {\user2{e}},\\ {{\dot{{\user2{g}}}}}&= d\left[ {\user2{e}}\times {\user2{E}}+\frac{1}{m}{\user2{e}}\times \left( {\user2{p}}\times {\user2{B}}\right) \right] +d\delta \left[ \frac{1}{I} ({\user2{e}}\cdot {\user2{B}}) \left( {\user2{g}}\times {\user2{e}}\right) +{\user2{e}}\times \left( {\user2{E}}'({\user2{r}}){\user2{e}}\right) +\frac{1}{m}{\user2{e}}\times \left( {\user2{p}}\times {\user2{B}}'({\user2{r}}){\user2{e}}\right) \right] . \end{aligned} \end{aligned}$$

In Appendix 2, we show that this system can be derived from an appropriate approximated Lagrange function given in (112). General properties of equations (42) in the special case of uniform and stationary electromagnetic fields were analysed in^4^. In this case these equations can be reduced to a Hamiltonian system with two degrees of freedom which is non-integrable except two cases with specific values of parameters. Also results of numerical simulations for a water molecule and CdS nanocrystal physical dipoles were shown.

Application of equations (42) to the special case of non-constant electromagnetic fields is given in our previous article^5^. Namely, in this paper we analysed numerically the dynamics of a small dipole in the superposition of quadrupolar magnetic field ${\user2{B}}({\user2{r}})=\tfrac{B_1}{2}(-x,-y,2z)$ and electric field ${\user2{E}}({\user2{r}})=E_z{\user2{e}}_z-\nabla \Phi _3{\user2{r}})$, with sextupole potential $\Phi _3=\tfrac{U_0}{8D^3}\left( z^3-\tfrac{3}{2}z(x^2+y^2)\right)$, where $U_0$ is the trapping voltage and *D* is the characteristic trap size. We demonstrated that such a non-homogeneous electromagnetic field seems to be useful for trapping of polar particles. We shown this by numerical calculations of trajectories of the centre of mass various physical polar particles: the DAST nanocrystal, the cellulose nanocrystal and protein *lac* repressor. Simulations with different initial conditions give different mass centre trajectories but they remain bounded to a restricted volume. However the general properties of equations (42) have not been analyzed so far, and filling this gap is the goal of this article. We hope that this analysis enables a better understanding of the complex dynamics of the dipole in non-homogeneous electromagnetic fields and it will help for example in the construction of new electromagnetic traps for polar particles.

System (42) has two geometric first integrals

43 $$\begin{aligned} H_0= {\user2{e}}\cdot {\user2{e}}, \qquad H_1={\user2{e}}\cdot {\user2{g}}\end{aligned}$$

and the energy integral

44 $$\begin{aligned} H = \frac{1}{2m} {\user2{p}}\cdot {\user2{p}}+ \frac{1}{2I}{\user2{g}}\cdot {\user2{g}}- d{\user2{e}}\cdot {\user2{E}}-\frac{d\delta }{2}{\user2{e}}\cdot \left( {\user2{E}}'({\user2{r}}){\user2{e}}\right) . \end{aligned}$$

Checking that *H* is a first integral needs some efforts. Namely, during calculations we have to use the following identities

$$\begin{aligned} \begin{aligned}{}&2{\user2{g}}\cdot \left( {\user2{e}}\times {\user2{E}}'({\user2{r}}){\user2{e}}\right) -({\user2{g}}\times {\user2{e}}) \cdot {\user2{E}}'({\user2{r}}){\user2{e}}-{\user2{e}}\cdot {\user2{E}}'({\user2{r}})\left( {\user2{g}}\times {\user2{e}}\right) =0,\\&{\user2{p}}\cdot {\user2{E}}''({\user2{r}})\left( {\user2{e}},{\user2{e}}\right) -{\user2{e}}\cdot {\user2{E}}''({\user2{r}})\left( {\user2{p}},{\user2{e}}\right) =0,\quad {\user2{p}}\cdot \left( ({\user2{g}}\times {\user2{e}})\times {\user2{X}}\right) +{\user2{g}}\cdot \left( {\user2{e}}\times ({\user2{p}}\times {\user2{X}})\right) =0. \end{aligned} \end{aligned}$$

In the last formula ${\user2{X}}={\user2{B}}$ or ${\user2{X}}={\user2{B}}'({\user2{r}}){\user2{e}}$. All of them can be checked directly and we left it to the reader.

Let *F* and *G* be two smooth functions defined on $M^{12}=(\mathbb {R}^3)^4=\mathbb {R}^{12}$ with coordinates ${\user2{x}}=({\user2{r}},{\user2{p}}, {\user2{e}}, {\user2{g}})$. We define their bracket as

45 $$\begin{aligned} \{F,G\}({\user2{x}})= & {} \dfrac{\partial F }{\partial {\user2{x}}}^T {\user2{J}}({\user2{x}}) \dfrac{\partial G }{\partial {\user2{x}}},\qquad \text {where}\end{aligned}$$

46 $$\begin{aligned} {\user2{J}}({\user2{x}})= & {} \begin{bmatrix} \varvec{0}&{} {\mathrm {Id}}_3 &{} \varvec{0}&{} \varvec{0}\\ -{\mathrm {Id}}_3 &{} {{\widehat{{\user2{b}}}}} &{} \varvec{0}&{} \varvec{\Omega }\\ \varvec{0}&{} \varvec{0}&{}\varvec{0}&{} -{{\widehat{{\user2{e}}}}} \\ \varvec{0}&{} -\varvec{\Omega }^T &{} -{\widehat{{\user2{e}}}} &{} -d \delta ({\user2{e}}\cdot {\user2{B}}){{\widehat{{\user2{e}}}}} -{{\widehat{{\user2{g}}}}} \end{bmatrix}, \end{aligned}$$

with

$$\begin{aligned} \begin{aligned} {\user2{b}}&= -d{\user2{B}}'({\user2{r}}){\user2{e}}-\frac{d\delta }{2} {\user2{B}}''({\user2{r}})({\user2{e}},{\user2{e}}), \qquad \varvec{\Omega }=d [ {\user2{e}}{\user2{B}}^{T}({\user2{r}}) - ({\user2{e}}\cdot {\user2{B}}({\user2{r}})){\mathrm {Id}}_3 ]+d\delta [ {\user2{e}}\left( {\user2{B}}'({\user2{r}}){\user2{e}}\right) ^{T} - \left( {\user2{e}}\cdot \left( {\user2{B}}'({\user2{r}}){\user2{e}}\right) \right) {\mathrm {Id}}_3 ]. \end{aligned} \end{aligned}$$

We have the following.

### Lemma 3.1

*The bracket defined by* (45) *is bilinear, antisymmetric and satisfies the Leibniz identity. Moreover, by Gauss’s law for magnetism* $\nabla \cdot {\user2{B}}=\varvec{0},$* it satisfies the Jacobi identity and thus it defines a degenerated Poisson structure of rank 10 for which functions* $H_0$ *and* $H_1$ *are Casimir functions.*

### Proof

Except the Jacobi identity all statements of the lemma are easy to check. To prove the Jacobi identity we proceed like in Lemma 2.1. However, we have to use the fact that from Gauss’s law for magnetism $\nabla \cdot {\user2{B}}=\varvec{0}$ it follows that all first and second partial derivatives of the left hand side of this condition also vanish identically. $\square$

Now, we can state the main result in this section.

### Theorem 3.2

*Equations* (42) *are Hamiltonian with respect to the Poisson bracket* (45) *and Hamilton’s function* *H* *given in* (44).

A direct proof of this theorem we left to the reader.

We showed that system (42) restricted to level

47 $$\begin{aligned} {{\mathcal {M}}}=\left\{ {({\user2{r}},{\user2{p}},{\user2{e}}, {\user2{g}})\in M^{12} }\;\vert \;\; {{\user2{e}}\cdot {\user2{e}}=1, \quad {\user2{e}}\cdot {\user2{g}}=0} \,\right\} \end{aligned}$$

is Hamiltonian with five degrees of freedom. It has one first integral *H*. Hence, for its integrability four additional commuting first integral are needed.

## First integrals

It seems that for general form of ${\user2{E}}({\user2{r}})$ and ${\user2{B}}({\user2{r}})$ the only first integrals of system (42) are *H*, $H_{0}$ and $H_1$. In this section, we look for cases when the system admits an additional first integral which is polynomial of low degree with respect to momenta ${\user2{p}}$ and ${\user2{g}}$. However, even for the case of first integrals which are linear with respect to ${\user2{p}}$ and ${\user2{g}}$, without additional restrictions concerning coefficients of such a polynomial the problem is not tractable.

Thus, we limited ourselves to searching of first integral *F* linear in variables ${\user2{p}}$, ${\user2{g}}$, and ${\user2{e}}$ with coefficients which are functions of ${\user2{r}}$, that is

48 $$\begin{aligned} F=\varvec{P}({\user2{r}})\cdot {\user2{p}}+{\user2{Q}}({\user2{r}})\cdot {\user2{e}}+{\user2{R}}({\user2{r}})\cdot {\user2{g}}. \end{aligned}$$

Equating to zero Lie derivative we obtain a system of 62 partial differential equations for these unknown coefficients $\varvec{P}({\user2{r}})$, ${\user2{Q}}({\user2{r}})$ and ${\user2{R}}({\user2{r}})$, as well as for the electric and magnetic fields ${\user2{E}}({\user2{r}})$ and ${\user2{B}}({\user2{r}})$. Fortunately partial differential equations for coefficients $\varvec{P}({\user2{r}})$ and ${\user2{R}}({\user2{r}})$ separate from remaining equations. Solving them we obtain that ${\user2{R}}({\user2{r}})={\user2{R}}$ is a constant vector, and

49 $$\begin{aligned} \varvec{P}({\user2{r}}) = {\user2{C}}+ \varvec{D}\times {\user2{r}}, \qquad {\user2{Q}}({\user2{r}}) = d\varvec{P}({\user2{r}})\times {\user2{B}}({\user2{r}}), \qquad {\user2{C}}, \varvec{D}\in \mathbb {R}^3. \end{aligned}$$

Additionally, the following equation

50 $$\begin{aligned} \left( {\user2{R}}-\varvec{D}\right) \times {\user2{B}}({\user2{r}})=\varvec{0}, \end{aligned}$$

relating constant vectors ${\user2{R}}$, ${\user2{C}}$, and $\varvec{D}$ with the magnetic field ${\user2{B}}({\user2{r}})$ has to be fulfilled. This equation has two kinds of solutions. Either $\varvec{D}={\user2{R}}$ and ${\user2{B}}({\user2{r}})$ is arbitrary, or $\varvec{D}\ne {\user2{R}}$ and ${\user2{B}}({\user2{r}})=b({\user2{r}}) ( {\user2{R}}-\varvec{D})$ where $b({{\mathbf {r}}})$ is an arbitrary smooth function. We show that only first case can occurs.

### Lemma 4.1

*Assume that the system has a first integral of the form* (48) *with coefficients* $\varvec{P}({\user2{r}})$, ${\user2{Q}}({\user2{r}})$ *and* ${\user2{R}}({\user2{r}})$ *satisfying the above conditions* (49) *and* (50). *If* ${\user2{B}}({\user2{r}})\ne 0$*, then* $\varvec{D}={\user2{R}}$.

### Proof

Assume that $\varvec{D}\ne {\user2{R}}$. Then ${\user2{B}}({\user2{r}})=b({\user2{r}}) ( {\user2{R}}-\varvec{D})$, where ${\user2{b}}({\user2{r}})\ne 0$, and the first integral has the following form

51 $$\begin{aligned} F =\left( {\user2{C}}+\varvec{D}\times {\user2{r}}\right) \cdot \left( {\user2{p}}-d{\user2{e}}\times {\user2{B}}({\user2{r}})\right) + {\user2{R}}\cdot {\user2{g}}, \qquad {\user2{B}}({\user2{r}})=b({\user2{r}}) ( {\user2{R}}-\varvec{D}). \end{aligned}$$

The Lie derivative of *F* given above is a polynomial of third degree in ${\user2{p}}$, ${\user2{e}}$ and ${\user2{g}}$ of the following form

52 $$\begin{aligned} {\frac{\mathrm {d}}{\mathrm {d}t}}F=dR_{010}+\frac{d}{m}R_{110}+\frac{d\delta }{2}R_{020}+ \frac{d\delta }{2m}R_{120}+\frac{d\delta }{I}R_{021}, \end{aligned}$$

where $R_{ijk}$ are homogeneous polynomials of variables $({\user2{p}},{\user2{e}},{\user2{g}})$, of total degree $i+j+k$. Clearly, *F* is a first integral if and only if all $R_{ijk}$ vanishes identically. Term $R_{1,1,0}$ is a bilinear form $R_{1,1,0}= {\user2{e}}^T {\user2{M}}{\user2{p}}$ with matrix ${\user2{M}}$ given by

53 $$\begin{aligned} {\user2{M}}=\left( ({\user2{C}}+\varvec{D}\times {\user2{r}})\times {\user2{T}}\right) \nabla b({\user2{r}})^T -\nabla b({\user2{r}})\left( ({\user2{C}}+\varvec{D}\times {\user2{r}})\times {\user2{T}}\right) ^T -b({\user2{r}}){\widehat{{\user2{T}}}}{\widehat{\varvec{D}}} +b({\user2{r}}){\widehat{{\user2{R}}}}{\widehat{{\user2{T}}}} , \end{aligned}$$

where ${\user2{T}}={\user2{R}}-\varvec{D}$. Diagonal elements of this matrix are following

54 $$\begin{aligned} M_{ii} = -b({\user2{r}})(T_j^2 +T_k^2) \qquad (i,j,k)=(1,2,3), (3,1,2), (2,3,1). \end{aligned}$$

Thus ${\user2{T}}=\varvec{0}$, and so $\varvec{D}={\user2{R}}$. This is a contradiction which finishes the proof. $\square$

For further consideration we assume that ${\user2{B}}({\user2{r}})\ne 0$. So, by the above lemma, if the first integral (48) exists than it has the form

55 $$\begin{aligned} F=\left( {\user2{C}}+{\user2{R}}\times {\user2{r}}\right) \cdot \left( {\user2{p}}-d{\user2{e}}\times {\user2{B}}({\user2{r}})\right) + {\user2{R}}\cdot {\user2{g}}. \end{aligned}$$

As we will see it later vector ${\user2{S}}({\user2{r}}):= {\user2{C}}+{\user2{R}}\times {\user2{r}}$ plays an important role.

The Lie derivative of *F* is a polynomial of third degree in variables $({\user2{p}},{\user2{e}},{\user2{g}})$ of the form given by (52) where polynomials $R_{ijk}$ have the forms

56a $$\begin{aligned} R_{010}&={\user2{S}}({\user2{r}})\cdot {\user2{E}}'({\user2{r}}){\user2{e}}+ {\user2{R}}\cdot \left( {\user2{e}}\times {\user2{E}}({\user2{r}})\right) , \end{aligned}$$

56b $$\begin{aligned} R_{110}&={\user2{S}}({\user2{r}})\cdot \left( {\user2{p}}\times {\user2{B}}'({\user2{r}}){\user2{e}}\right) - {\user2{S}}({\user2{r}})\cdot \left( {\user2{e}}\times {\user2{B}}'({\user2{r}}){\user2{p}}\right) -({\user2{R}}\times {\user2{p}})\cdot ({\user2{e}}\times {\user2{B}}({\user2{r}}))+ {\user2{R}}\cdot \left( {\user2{e}}\times ({\user2{p}}\times {\user2{B}}({\user2{r}}))\right) , \end{aligned}$$

56c $$\begin{aligned} R_{020}&={\user2{S}}({\user2{r}})\cdot {\user2{E}}''({\user2{r}})({\user2{e}},{\user2{e}})+ 2{\user2{R}}\cdot \left( {\user2{e}}\times {\user2{E}}'({\user2{r}}){\user2{e}}\right) , \end{aligned}$$

56d $$\begin{aligned} R_{120}&={\user2{S}}({\user2{r}})\cdot \left( {\user2{p}}\times {\user2{B}}''({\user2{r}})({\user2{e}},{\user2{e}})\right) + 2{\user2{R}}\cdot \left( {\user2{e}}\times ({\user2{p}}\times {\user2{B}}'({\user2{r}}){\user2{e}})\right) ,\end{aligned}$$

56e $$\begin{aligned} R_{021}&={\user2{S}}({\user2{r}})\cdot \left( ({\user2{g}}\times {\user2{e}})\times {\user2{B}}'({\user2{r}}){\user2{e}}\right) + ({\user2{e}}\cdot {\user2{B}}({\user2{r}}))\left( {\user2{R}}\cdot ({\user2{g}}\times {\user2{e}})\right) . \end{aligned}$$

Now, analysing equations $R_{ijk}=0$ we can deduce additional necessary conditions for the existence of first integral *F*.

### Lemma 4.2

*If* (55) *is a first integral of system* (42)*, then*

57 $$\begin{aligned} {\user2{E}}'({\user2{r}}){\user2{S}}({\user2{r}}) -{\user2{R}}\times {\user2{E}}({\user2{r}})=\varvec{0}, \qquad {\user2{B}}'({\user2{r}}){\user2{S}}({\user2{r}}) -{\user2{R}}\times {\user2{B}}({\user2{r}})=\varvec{0}. \end{aligned}$$

### Proof

We deduce these two necessary conditions from equations $$R_{010}=0$$ and $$ R_{110}=0$$. Term $$R_{010}$$, see (56a), can be rewritten as

$$\begin{aligned} R_{010}=\Big ({\user2{E}}'({\user2{r}}) {\user2{S}}({\user2{r}})+{\user2{E}}({\user2{r}})\times {\user2{R}}\Big )\cdot {\user2{e}}, \end{aligned}$$

where we used symmetry of ${\user2{E}}'({\user2{r}})$. As $R_{010}=0$ for an arbitrary ${\user2{e}}$ we get first condition in (57).

Now we rewrite four terms of $R_{110}$, see (56b) in the following way

$$\begin{aligned} & {\user2{S}}(\user2{r})\cdot \left( \user2{p}\times \user2{B}'(\user2{r})\user2{e}\right) =\left( \user2{S}(\user2{r})\times \user2{p}\right) \cdot (\user2{B}'(\user2{r})\user2{e}) = (\widehat{\user2{S}(\user2{r})}\user2{p})\cdot (\user2{B}'(\user2{r})\user2{e})=-\user2{p}\cdot (\widehat{\user2{S}(\user2{r})}\user2{B}'(\user2{r})\user2{e}),\\&-\user2{S}(\user2{r})\cdot \left( \user2{e}\times \user2{B}'(\user2{r})\user2{p}\right) = -(\user2{B}'(\user2{r})\user2{p})\cdot \left( \user2{S}(\user2{r})\times \user2{e}\right) = -\user2{p}\cdot \left( \user2{B}'(\user2{r})^T\widehat{\user2{S}(\user2{r})}\user2{e}\right) ,\\&-(\user2{R}\times \user2{p})\cdot (\user2{e}\times \user2{B}(\user2{r})) =-({\widehat{\user2{R}}}\user2{p})\cdot (-\widehat{\user2{B}(\user2{r})}\user2{e})= -\user2{p}\cdot ({\widehat{\user2{R}}}\widehat{\user2{B}(\user2{r})}\user2{e}),\\&\user2{R}\cdot \left( \user2{e}\times (\user2{p}\times \user2{B}(\user2{r}))\right) = -(\user2{B}(\user2{r})\times \user2{p})\cdot (\user2{R}\times \user2{e})= -(\widehat{\user2{B}(\user2{r})}\user2{p})\cdot ({\widehat{\user2{R}}}\user2{e})= - \user2{p}\cdot (\widehat{\user2{B}(\user2{r})}^T{\widehat{\user2{R}}}\user2{e}). \end{aligned} $$

Thus, $$R_{110}=-{\user2{p}}\cdot \varvec{U}{\user2{e}}$$, where

58 $$\begin{aligned} \varvec{U}={{\widehat{{\user2{S}}}}}({\user2{r}}) {\user2{B}}'({\user2{r}})+ {\user2{B}}'({\user2{r}})^T\widehat{{\user2{S}}({\user2{r}})} +{\widehat{{\user2{R}}}}\widehat{{\user2{B}}({\user2{r}})} + \widehat{{\user2{B}}({\user2{r}})}^T{\widehat{{\user2{R}}}}. \end{aligned}$$

Using property (8), and facts that ${\text {Tr}}{\user2{B}}'({\user2{r}})=\nabla \cdot {\user2{B}}({\user2{r}})=0$ and ${\text {Tr}}{{\widehat{{\user2{R}}}}}=0$, we rewrite this matrix in the form

59 $$\begin{aligned}  U = \widehat{{B^{\prime}(r)S(r)}} + \widehat{{B(r) \times R}}  \end{aligned}$$

As $R_{110}=-{\user2{p}}\cdot \varvec{U}{\user2{e}}=0$ for arbitrary ${\user2{p}}$ and arbitrary ${\user2{e}}$, the antisymmetric matrix $\varvec{U}$ vanishes, and thus, also the corresponding vector

60 $$\begin{aligned} {\user2{B}}'({\user2{r}}) {\user2{S}}({\user2{r}}) + {\user2{B}}({\user2{r}})\times {\user2{R}}=\varvec{0}. \end{aligned}$$

This ends the proof. $\square$

For further considerations it is important to notice the following.

### Corollary 4.3

*Conditions* (57) *can be written in the following form*

61 $$\begin{aligned} \left[ {\user2{S}}({\user2{r}}), {\user2{E}}({\user2{r}}) \right] =\varvec{0}\quad \text {and}\quad \left[ {\user2{S}}({\user2{r}}), {\user2{B}}({\user2{r}}) \right] =\varvec{0}, \end{aligned}$$

*where* $[\cdot , \cdot ]$ *denotes the commutator of vector fields.*

The commutator $\left[ {\user2{X}}({\user2{r}}) , \varvec{Y}({\user2{r}})\right]$ of arbitrary vector fields ${\user2{X}}({\user2{r}}),\varvec{Y}({\user2{r}})$ in $\mathbb {R}^3$ means the Lie bracket of vector fields defined as

$$\begin{aligned} \left[ {\user2{X}}({\user2{r}}) , \varvec{Y}({\user2{r}})\right] =\varvec{Y}'({\user2{r}}){\user2{X}}(r)- {\user2{X}}'({\user2{r}})\varvec{Y}({\user2{r}}), \end{aligned}$$

where ${\user2{X}}'({\user2{r}})$ and $\varvec{Y}'({\user2{r}})$ mean Jacobian matrices $\partial _j X_i$ and $\partial _j Y_i$ for $i,j=1,2,3$.

### Proof

When we substitute in the above definition ${\user2{X}}({\user2{r}})={\user2{S}}(r)$ and notice that ${\user2{X}}'({\user2{r}})={\user2{S}}'({\user2{r}})={{\widehat{{\user2{R}}}}}$, then we can rewrite this expression as

$$\begin{aligned} \left[ {\user2{S}}({\user2{r}}) , \varvec{Y}({\user2{r}})\right] =\varvec{Y}'({\user2{r}}){\user2{S}}({\user2{r}})- {\user2{R}}\times \varvec{Y}({\user2{r}}), \end{aligned}$$

and this ends the proof. $\square$

In other words, if the system admits a first integral (55), then ${\user2{S}}({\user2{r}})$ is a symmetry field of electric and magnetic fields ${\user2{E}}({\user2{r}})$ and ${\user2{B}}({\user2{r}})$. This property gives strong restrictions on these fields. Moreover, if $\delta =0$, then all the conditions are fulfilled. So, we can summarize the above considerations with the following.

### Theorem 4.4

*Assume that*${\user2{B}}({\user2{r}})\ne 0$*and*$\delta =0$. *Then the system* (42) *has integral of the type* (48) *if and only if it has the form* (55) *and vector fields* ${\user2{E}}({\user2{r}})$ *and* ${\user2{B}}({\user2{r}})$ *commute with* ${\user2{S}}({\user2{r}})$.

For $\delta \ne 0$ we have three additional conditions $R_{020}=0$, $R_{120}=0$ and $R_{021}=0$. The term $R_{020}$ can be rewritten as

$$\begin{aligned} R_{020}={\user2{e}}\cdot \varvec{G}{\user2{e}}, \end{aligned}$$

where

$$\begin{aligned} \varvec{G}:={\user2{S}}({\user2{r}})\cdot {\user2{E}}''({\user2{r}})-2{\widehat{{\user2{R}}}}{\user2{E}}'({\user2{r}}). \end{aligned}$$

Condition $$ R_{020}=0$$ implies that the symmetric part of matrix $$\varvec{G}$$ vanishes and this is equivalent to the following equality

62 $$\begin{aligned} {\user2{S}}({\user2{r}})\cdot {\user2{E}}''({\user2{r}}) + \left[ {\user2{E}}'({\user2{r}}),{\widehat{{\user2{R}}}} \right] =0. \end{aligned}$$

Polynomials $R_{021}$ and $R_{120}$ can be rewritten as

$$\begin{aligned} \begin{aligned} R_{021}&=\Big ( {\user2{e}}\times \left[ {\user2{B}}'({\user2{r}}){\user2{e}}\times {\user2{S}}({\user2{r}}) \right] + ({\user2{e}}\cdot {\user2{B}}({\user2{r}})) {\user2{e}}\times {\user2{R}}\Big )\cdot {\user2{g}},\\ R_{120}&=\Big ({\user2{B}}''({\user2{r}})({\user2{e}},{\user2{e}})\times {\user2{S}}({\user2{r}})+ 2({\user2{B}}'({\user2{r}}){\user2{e}})\times ({\user2{R}}\times {\user2{e}})\Big )\cdot {\user2{p}}. \end{aligned} \end{aligned}$$

Thus, conditions $R_{021}=0$ and $R_{120}=0$ and are equivalent to

63a $$\begin{aligned}&{\user2{e}}\times \left[ {\user2{B}}'({\user2{r}}){\user2{e}}\times {\user2{S}}({\user2{r}}) \right] + ({\user2{e}}\cdot {\user2{B}}({\user2{r}}) ){\user2{e}}\times {\user2{R}}=\varvec{0}, \end{aligned}$$

63b $$\begin{aligned}{}&{\user2{B}}''({\user2{r}})({\user2{e}},{\user2{e}})\times {\user2{S}}({\user2{r}})+ 2({\user2{B}}'({\user2{r}}){\user2{e}})\times ({\user2{R}}\times {\user2{e}}) =\varvec{0}, \end{aligned}$$

respectively. Each of these conditions defines three quadratic forms in components of ${\user2{e}}$, and all of them have to vanish. So, altogether we have 36 additional conditions when $\delta \ne 0$.

Let us analyse the above conditions for given vectors ${\user2{C}}$ and ${\user2{R}}$. They have form of a system of partial differential equations which must be fulfilled by fields ${\user2{E}}({\user2{r}})$ and ${\user2{B}}({\user2{r}})$. We consider three complementary cases.

**L1**: Assume that ${\user2{R}}=\varvec{0}$ and ${\user2{C}}\ne \varvec{0}$. By a proper orientation of the inertial frame we can achieve that ${\user2{C}}=(0,0,C_3)$, then by a rescaling we obtain that $C_3=1$. In effect in this case we will assume that ${\user2{C}}=(0,0,1)$.
**L2**: Assume that ${\user2{R}}\ne \varvec{0}$ and ${\user2{C}}=\varvec{0}$. By the same arguments as in the previous case we will assume that ${\user2{R}}=(0,0,1)$.
**L3**: Assume that ${\user2{R}}\ne \varvec{0}$ and ${\user2{C}}\ne \varvec{0}$. By a proper orientation of the inertial frame we can achieve that ${\user2{R}}=(0,0,1)$, and ${\user2{C}}=(0,C_2,C_3)$. A translation ${\user2{r}}\rightarrow {\user2{r}}+ {\user2{a}}$ transforms ${\user2{C}}\rightarrow {\user2{C}}+ {\user2{R}}\times {\user2{a}}$. Taking ${\user2{a}}=(0,-C_2,0)$ we obtain ${\user2{C}}=(0,0,C_3)$. So, in this case we will assume that ${\user2{R}}=(0,0,1)$ and ${\user2{C}}=(0,0,c)$, $c\in \mathbb {R}$.

In each case we perform analysis into two steps. First, we give necessary conditions which guarantee that in (52) terms $R_{010}$ and $R_{110}$ vanish. That is, by Lemma 4.2, we find ${\user2{E}}({\user2{r}})$ and ${\user2{B}}({\user2{r}})$ which satisfy (57). If $\delta =0$, then these conditions are also sufficient for the existence of first integral (55). In the second step we assume that ${\user2{E}}({\user2{r}})$ and ${\user2{B}}({\user2{r}})$ satisfy derived conditions and we find those which satisfy $R_{020}=0$, $R_{210}=0$ and $R_{012}=0$, or, equivalently, which satisfy conditions (62), (63a) and (63b), respectively.

### Lemma 4.5

*Assume that the system* (42) *admits a first integral of the form* (55) *and case* **L1** *occurs. Then*

64 $$\begin{aligned} {\user2{E}}({\user2{r}})=(E_1(x,y), E_2(x,y), E_3), \qquad \dfrac{\partial E_2 }{\partial x}(x,y) = \dfrac{\partial E_1 }{\partial y}(x,y) , \end{aligned}$$

*and*

65 $$\begin{aligned} {\user2{B}}({\user2{r}})=(B_1(x,y,), B_2(x,y), B_3(x,y)), \qquad \dfrac{\partial B_1 }{\partial x}(x,y) = -\dfrac{\partial B_2 }{\partial y}(x,y). \end{aligned}$$

### Proof

If the system admits first integral (55), then by Lemma 4.2, conditions (57) are fulfilled. For ${\user2{R}}=(0,0,0)$ they read ${\user2{E}}'({\user2{r}}){\user2{C}}=\varvec{0}$ and ${\user2{B}}'({\user2{r}}){\user2{C}}=\varvec{0}$. As, by assumption, ${\user2{C}}=(0,0,1)$ they give

$$\begin{aligned} \dfrac{\partial {\user2{E}} }{\partial z}({\user2{r}})= \dfrac{\partial {\user2{B}} }{\partial z}({\user2{r}})=0. \end{aligned}$$

Now, the assumption $\nabla {\user2{E}}({\user2{r}})\times {\user2{R}}=\varvec{0}$ implies that $E_3$ is a constant function and that the second equality in (64) is fulfilled. The assumption $\nabla \cdot {\user2{B}}({\user2{r}})=0$ gives the second equality in (65). $\square$

### Lemma 4.6

*Assume that the system* (42) *admits a first integral of the form* (55) *and case* **L1** *occurs. If* $\delta \ne 0$*, then* ${\user2{E}}({\user2{r}})$ *is given by* (64) *and*

66 $$\begin{aligned} {\user2{B}}({\user2{r}})=(B_1, B_2, B_3(x,y)), \end{aligned}$$

*where* $B_1$ *and* $B_2$ *are constant.*

### Proof

We can assume that fields ${\user2{E}}({\user2{r}})$ and ${\user2{B}}({\user2{r}})$ have the form given by the previous lemma. For ${\user2{R}}=\varvec{0}$ and ${\user2{C}}=(0,0,1)$ condition (62) reads $E_3''({\user2{r}})=0$, but it is fulfilled because by the remark before this lemma $E_3({\user2{r}})$ is a constant function.

For the above choice of ${\user2{C}}$ and ${\user2{R}}$ condition $R_{021}=0$, see (63a), reads

67 $$\begin{aligned} e_3 {\user2{B}}'({\user2{r}}){\user2{e}}- (0,0,{\user2{e}}\cdot {\user2{B}}'({\user2{r}}){\user2{e}})=0 \end{aligned}$$

and it implies that $B_1({\user2{r}})=B_1$ and $B_2({\user2{r}})=B_2$ are constant functions.

Condition $R_{120}=0$, see (63b), has the form

68 $$\begin{aligned} {\user2{B}}''({\user2{r}})({\user2{e}},{\user2{e}})\times (0,0,1) = (B_2''({\user2{r}})({\user2{e}},{\user2{e}}), -B_1''({\user2{r}})({\user2{e}},{\user2{e}}),0)=(0,0,0) \end{aligned}$$

and it does not give new restrictions on ${\user2{B}}({\user2{r}})$ because we already proved that $B_1({\user2{r}})$ and $B_2({\user2{r}})$ are constant functions. This ends the proof. $\square$

An analysis of remaining case is convenient to perform in cylindrical coordinates $(\rho ,\varphi ,z)$ in which

69 $$\begin{aligned} {\user2{r}}= \rho {\user2{e}}_{\rho } + z{\user2{e}}_z, \qquad {\user2{e}}_{\rho } =(\cos \varphi , \sin \varphi ,0), \quad {\user2{e}}_{\varphi }= (-\sin \varphi ,\cos \varphi ,0), \quad {\user2{e}}_z=(0,0,1). \end{aligned}$$

For an arbitrary vector ${\user2{X}}=(X_1,X_2,X_3)\in \mathbb {R}^3$ we have

70 $$\begin{aligned} X_1 = X_{\rho } \cos \varphi + X_{\varphi } \sin \varphi , \qquad X_2 = -X_{\rho } \sin \varphi + X_{\varphi } \cos \varphi \qquad X_3 = X_z. \end{aligned}$$

Now, let us define

71 $$\begin{aligned} \varvec{Y}= {\user2{X}}'({\user2{r}})({\user2{C}}+ {\user2{R}}\times {\user2{r}}) -{\user2{R}}\times {\user2{X}}({\user2{r}}). \end{aligned}$$

Then cylindrical components of this vector are following

72 $$\begin{aligned} \begin{aligned} Y_{\rho }&= \frac{zR_{\rho } -C_{\varphi }}{\rho }X_{\varphi } -R_{\varphi }X_z + (C_\rho +z R_{\varphi }) \dfrac{\partial X_{\rho } }{\partial \rho } + \frac{ C_{\varphi }-zR_{\rho } +\rho R_z}{\rho } \dfrac{\partial X_{\rho } }{\partial \varphi }+ (C_z -\rho R_{\varphi }) \dfrac{\partial X_{\rho } }{\partial z}, \\ Y_{\varphi }&= \frac{C_{\varphi }-z R_{\rho }}{\rho }X_{\rho } + R_{\rho }X_z + (C_\rho +z R_{\varphi }) \dfrac{\partial X_{\varphi } }{\partial \rho } + \frac{ C_{\varphi }-zR_{\rho } +\rho R_z}{\rho } \dfrac{\partial X_{\varphi } }{\partial \varphi }+ (C_z -\rho R_{\varphi }) \dfrac{\partial X_{\varphi } }{\partial z}, \\ Y_z&= R_{\varphi }X_{\rho } - R_{\rho }X_\varphi + (C_\rho +z R_{\varphi }) \dfrac{\partial X_z }{\partial \rho } + \frac{ C_{\varphi }-zR_{\rho } +\rho R_z}{\rho } \dfrac{\partial X_z }{\partial \varphi }+ (C_z -\rho R_{\varphi }) \dfrac{\partial X_z }{\partial z}. \end{aligned} \end{aligned}$$

Notice that if $(C_{\rho }, C_{\varphi }, C_z)=(0,0,0)$ and $(R_{\rho }, R_{\varphi }, R_z)=(0,0,1)$, then

73 $$\begin{aligned} (Y_{\rho }, Y_{\varphi }, Y_z)= \left( \dfrac{\partial X_{\rho } }{\partial \varphi }, \dfrac{\partial X_{\varphi } }{\partial \varphi }, \dfrac{\partial X_z }{\partial \varphi } \right) . \end{aligned}$$

Moreover, if $(C_{\rho }, C_{\varphi }, C_z)=(0,0,c)$ and $(R_{\rho }, R_{\varphi }, R_z)=(0,0,1)$, then

74 $$\begin{aligned} (Y_{\rho }, Y_{\varphi }, Y_z)=\left( \dfrac{\partial X_{\rho } }{\partial \varphi } +c\dfrac{\partial X_{\rho } }{\partial z} , \dfrac{\partial X_{\varphi } }{\partial \varphi } +c\dfrac{\partial X_{\varphi } }{\partial z} , \dfrac{\partial X_z }{\partial \varphi } +c \dfrac{\partial X_z }{\partial \varphi } \right) . \end{aligned}$$

From the above calculations we have the following.

### Corollary 4.7

*Assume that*${\user2{C}}=\varvec{0}$*and*${\user2{R}}={\user2{e}}_z$. *Then*$\varvec{Y}=\varvec{0}$*if and only if*

75 $$\begin{aligned} X_{\sigma } = X_{\sigma }(\rho ,z) \quad \text {for}\quad \sigma \in \{\rho ,\varphi ,z\}. \end{aligned}$$

### Corollary 4.8

*Assume that*${\user2{C}}=c {\user2{e}}_z$*and*${\user2{R}}={\user2{e}}_z$*,*$c\in \mathbb {R}$. *Then*$\varvec{Y}=\varvec{0}$*if and only if*

76 $$\begin{aligned} X_{\sigma } = X_{\sigma }(\rho ,\zeta ) \quad \text {for}\quad \sigma \in \{\rho ,\varphi ,z\}, \end{aligned}$$

*where*$$\zeta =z-c\varphi $$.

Let us notice variables $(\rho ,\varphi ,\zeta)$ that appear in this corollary define the so-called helical coordinate system. This system is not orthogonal but it has various applications, also in electrodynamics, see, for example^10,11^.

### Lemma 4.9

*Assume that the system* (42) *admits a first integral of the form* (55) *and case* **L2** *occurs. Then*

77 $$\begin{aligned} {\user2{E}}({\user2{r}})=\begin{bmatrix} \cos (\varphi )E_\rho (\rho ,z) - \sin ( \varphi )E_\varphi (\rho ,z) \\ \sin (\varphi )E_\rho (\rho ,z) + \cos ( \varphi )E_\varphi (\rho ,z) \\ E_z(\rho ,z) \end{bmatrix}, \qquad E_{\varphi }=\frac{\alpha }{\rho }, \quad \dfrac{\partial E_{\rho } }{\partial z} = \dfrac{\partial E_z }{\partial \rho }, \quad \alpha \in \mathbb {R}\end{aligned}$$

*and*

78 $$\begin{aligned} {\user2{B}}({\user2{r}})=\begin{bmatrix} \cos (\varphi )B_\rho (\rho ,z) - \sin ( \varphi )B_\varphi (\rho ,z) \\ \sin (\varphi )B_\rho (\rho ,z) + \cos ( \varphi )B_\varphi (\rho ,z) \\ B_z(\rho ,z) \end{bmatrix}, \qquad \frac{1}{\rho } \frac{\partial }{\partial \rho }\left( \rho B_{\rho } \right) +\dfrac{\partial B_{z} }{\partial z}=0. \end{aligned}$$

### Proof

Let us substitute ${\user2{X}}({\user2{r}})={\user2{E}}({\user2{r}})$ in the formula (71). Then the first condition in (57) is equivalent to $\varvec{Y}=\varvec{0}$. As in the considered case ${\user2{C}}=\varvec{0}$ and ${\user2{R}}={\user2{e}}_z$, by Corollary 4.7 we have

$$\begin{aligned} \dfrac{\partial E_{\rho } }{\partial \varphi }=\dfrac{\partial E_{\varphi } }{\partial \varphi }= \dfrac{\partial E_z }{\partial \varphi } =0. \end{aligned}$$

Now condition $\nabla \times {\user2{E}}({\user2{r}})=\varvec{0}$ expressed in cylindrical coordinates gives

$$\begin{aligned} \dfrac{\partial E_{\varphi } }{\partial z} = 0, \qquad \dfrac{\partial E_{\rho } }{\partial z} - \dfrac{\partial E_z }{\partial \rho } =0\qquad \frac{1}{\rho }E_{\varphi }+\dfrac{\partial E_{\varphi } }{\partial \rho }=0. \end{aligned}$$

Hence

79 $$\begin{aligned} E_{\varphi }=\frac{\alpha }{\rho } \quad \text {and}\quad \dfrac{\partial E_{\rho } }{\partial z} = \dfrac{\partial E_z }{\partial \rho }, \qquad \alpha \in \mathbb {R}. \end{aligned}$$

This proves the lemma for vector field ${\user2{E}}({\user2{r}})$.

The form of ${\user2{B}}({\user2{r}})$ we deduce in a similar way. $\square$

### Lemma 4.10

*Assume that the system* (42) *admits a first integral of the form* (55) *and case* **L2** *occurs. If* $\delta \ne 0$*, then* ${\user2{E}}({\user2{r}})$ *is given by* (77) *and*

80 $$\begin{aligned} {\user2{B}}({\user2{r}})=( - \sin ( \varphi )B_\varphi (\rho ,z) , \cos ( \varphi )B_\varphi (\rho ,z) , 0). \end{aligned}$$

### Proof

We can assume ${\user2{E}}({\user2{r}})$ and ${\user2{B}}({\user2{r}})$ have the form prescribed in the previous lemma. For ${\user2{C}}=\varvec{0}$ and ${\user2{R}}={\user2{e}}_z$, condition $R_{020}=0$, see (56c) or (62), is fulfilled identically.

Condition $R_{120}=0$, see (56d) and (63b), expressed in cylindrical coordinates immediately implies that $B_{\rho }=B_z=0$. But then condition $R_{021}=0$, see (56e) and (63a), is also fulfilled identically. $\square$

### Lemma 4.11

*Assume that the system* (42) *admits a first integral of the form* (55) *and case* **L3** *occurs. Then*

81 $$\begin{aligned} {\user2{E}}({\user2{r}})=\begin{bmatrix} \cos (\varphi )E_\rho (\rho ,\zeta ) - \sin ( \varphi )E_\varphi (\rho ,\zeta ) \\ \sin (\varphi )E_\rho (\rho ,\zeta ) + \cos ( \varphi )E_\varphi (\rho ,\zeta ) \\ E_z(\rho ,\zeta ) \end{bmatrix}, \qquad \zeta = z - c\varphi , \end{aligned}$$

*and*

82 $$\begin{aligned} {\user2{B}}({\user2{r}})=\begin{bmatrix} \cos (\varphi )B_\rho (\rho ,\zeta ) - \sin ( \varphi )B_\varphi (\rho ,\zeta ) \\ \sin (\varphi )B_\rho (\rho ,\zeta ) + \cos ( \varphi )B_\varphi (\rho ,\zeta ) \\ B_z(\rho ,\zeta ) \end{bmatrix}. \end{aligned}$$

*Moreover*

83 $$\begin{aligned} E_{\varphi }(\rho ,\zeta )=\frac{\alpha - c E_z(\rho ,\zeta )}{\rho }, \quad \dfrac{\partial E_{\rho } }{\partial \zeta } (\rho ,\zeta ) = \dfrac{\partial E_z }{\partial \rho }(\rho ,\zeta ) , \quad \alpha \in \mathbb {R}\end{aligned}$$

*and*

84 $$\begin{aligned} \frac{1}{\rho }\frac{\partial }{\partial \rho }\left( \rho B_{\rho } \right) - \frac{c}{\rho }\dfrac{\partial B_{\varphi } }{\partial \zeta }+ \dfrac{\partial B_z }{\partial \zeta }=0. \end{aligned}$$

### Proof

Let us substitute ${\user2{X}}({\user2{r}})={\user2{E}}({\user2{r}})$ in the formula (71). Then the first condition in (57) is equivalent to $\varvec{Y}=\varvec{0}$. As in the considered case ${\user2{C}}=c{\user2{e}}_z$ and ${\user2{R}}={\user2{e}}_z$, by Corollary 4.8 we have

$$\begin{aligned} E_{\sigma } = E_{\sigma }(\rho ,z) \quad \text {for}\quad \sigma \in \{\rho ,\varphi ,z\}. \end{aligned}$$

By the same reasons we have alse

$$\begin{aligned} B_{\sigma } = B_{\sigma }(\rho ,z) \quad \text {for}\quad \sigma \in \{\rho ,\varphi ,z\}. \end{aligned}$$

Now, conditions $\nabla \times {\user2{E}}({\user2{r}})=\varvec{0}$ and $\nabla \cdot {\user2{B}}({\user2{r}})=0$ are equivalent to (83) and (84), respectively. $\square$

### Lemma 4.12

*Assume that the system* (42) *admits a first integral of the form* (55) *and case* **L3** *occurs. If* $\delta \ne 0$*, then* ${\user2{E}}({\user2{r}})$ *is given by* (81) *and* (83) *and*

85 $$\begin{aligned} {\user2{B}}({\user2{r}})=B_\varphi (\rho ,\zeta ) \left[ - \sin ( \varphi ) , \cos ( \varphi ), \frac{c}{\rho } \right] ^T. \end{aligned}$$

### Proof

We can assume ${\user2{E}}({\user2{r}})$ and ${\user2{B}}({\user2{r}})$ have the form prescribed in the previous lemma. For ${\user2{C}}=c {\user2{e}}_z$ and ${\user2{R}}={\user2{e}}_z$, condition $R_{020}=0$, see (56c) or (62), is fulfilled identically.

Condition $R_{120}=0$, see (56d) and (63b), expressed in cylindrical coordinates implies that

$$\begin{aligned} B_\rho (\rho ,\zeta ) = 0 \quad \text {and}\quad B_z (\rho ,\zeta ) = \frac{c}{\rho } B_\varphi (\rho ,\zeta ). \end{aligned}$$

Then condition $R_{021}=0$, see (56e) and (63a), is also fulfilled identically. $\square$

As it is well known, if a Hamiltonian system generated by Hamiltonian *H* has a first integral *F* then Hamiltonian vector fields ${\user2{X}}_H$ and ${\user2{X}}_F$ commute. For the system considered in this paper the Poisson structure is not linear, see (46). Thus, even a very simple first integral *F* can generate complicated non-linear vector field. Amazingly, the first integral *F* given in (55) generates Hamiltonian vector field ${\user2{X}}_F$ which is affine with respect to coordinates $({\user2{r}},{\user2{p}}, {\user2{e}}, {\user2{g}})$.

### Lemma 4.13

*IfFgiven by* (55) *is a first integral of the system* (42)*, then the Hamiltonian vector field* ${\user2{X}}_F$ *has the form*

86 $$\begin{aligned} {\user2{X}}_F:={\user2{J}}({\user2{x}})\nabla F = [{\user2{C}}+{\user2{R}}\times {\user2{r}},{\user2{R}}\times {\user2{p}},{\user2{R}}\times {\user2{e}},{\user2{R}}\times {\user2{g}}]^T. \end{aligned}$$

### Proof

The partial derivatives of function *F* with respect all variables are following

87 $$\begin{aligned} \begin{aligned} \frac{\partial F}{\partial {\user2{r}}}&= {\user2{p}}\times {\user2{R}}-d{\user2{B}}^{'T}({\user2{r}})\big (({\user2{C}}+{\user2{R}}\times {\user2{r}})\times {\user2{e}}\big )- d\big (({\user2{e}}\times {\user2{B}}({\user2{r}}))\times {\user2{R}}\big ),\\ \frac{\partial F}{\partial {\user2{p}}}&={\user2{C}}+{\user2{R}}\times {\user2{r}},\\ \frac{\partial F}{\partial {\user2{e}}}&=-d\big ({\user2{B}}({\user2{r}})\times ({\user2{C}}+{\user2{R}}\times {\user2{r}}) \big ),\\ \frac{\partial F}{\partial {\user2{g}}}&={\user2{R}}. \end{aligned} \end{aligned}$$

From definition the vector field generated by *F* is ${\user2{X}}_{F}=\varvec{P}({\user2{x}})\nabla F({\user2{x}})$. As

$$\begin{aligned} \nabla _{{\user2{x}}}F=\left[ \frac{\partial F}{\partial {\user2{r}}},\frac{\partial F}{\partial {\user2{p}}},\frac{\partial F}{\partial {\user2{e}}},\frac{\partial F}{\partial {\user2{g}}}\right] ^T, \end{aligned}$$

we also split components of vector ${\user2{X}}_F$ accordingly, that is we set ${\user2{X}}_F=({\user2{X}}_{F,{\user2{r}}},{\user2{X}}_{F,{\user2{p}}},{\user2{X}}_{F,{\user2{e}}},{\user2{X}}_{F,{\user2{g}}} )$. Using explicit form of ${\user2{J}}(x)$, see (46), we easily obtain

88 $$\begin{aligned} \begin{aligned} {\user2{X}}_{F,{\user2{r}}}&= {\mathrm {Id}}_3 \frac{\partial F}{\partial {\user2{p}}}={\user2{C}}+{\user2{R}}\times {\user2{r}},\\ {\user2{X}}_{F,{\user2{e}}}&= -{{\widehat{{\user2{e}}}}} \frac{\partial F}{\partial {\user2{g}}}=-{\user2{e}}\times {\user2{R}}. \end{aligned} \end{aligned}$$

It is more complicated to calculate ${\user2{X}}_{F,{\user2{r}}}$. We have

89 $$\begin{aligned} \begin{aligned} {\user2{X}}_{F,{\user2{p}}}&= -{\mathrm {Id}}_3\frac{\partial F}{\partial {\user2{r}}}+ {{\widehat{{\user2{b}}}}}\frac{\partial F}{\partial {\user2{p}}} +\varvec{\Omega }\frac{\partial F}{\partial {\user2{e}}}\\&=-{\user2{p}}\times {\user2{R}}+d{\user2{B}}^{'T}({\user2{r}})\left( ({\user2{C}}+{\user2{R}}\times {\user2{r}})\times {\user2{e}}\right) + d ({\user2{e}}\times {\user2{B}}({\user2{r}}))\times {\user2{R}}\\&\quad -\left( d{\user2{B}}'({\user2{r}}){\user2{e}}+\frac{d\delta }{2} {\user2{B}}''({\user2{r}})({\user2{e}},{\user2{e}})\right) \times ({\user2{C}}+{\user2{R}}\times {\user2{r}})\\&\quad +d [ {\user2{e}}{\user2{B}}^{T}({\user2{r}}){\user2{R}}- ({\user2{e}}\cdot {\user2{B}}({\user2{r}})){\user2{R}}]+d\delta [ {\user2{e}}\left( {\user2{B}}'({\user2{r}}){\user2{e}}\right) ^{T}{\user2{R}}- \left( {\user2{e}}\cdot \left( {\user2{B}}'({\user2{r}}){\user2{e}}\right) \right) {\user2{R}}]. \end{aligned} \end{aligned}$$

Now we regroup and simplify several terms. Notice that

$$\begin{aligned} ({\user2{e}}\times {\user2{B}}({\user2{r}}))\times {\user2{R}}+{\user2{e}}{\user2{B}}^{T}({\user2{r}}){\user2{R}}- ({\user2{e}}\cdot {\user2{B}}({\user2{r}})){\user2{R}}= ({\user2{e}}\times {\user2{B}}({\user2{r}}))\times {\user2{R}}+ ({\user2{R}}\times {\user2{e}})\times {\user2{B}}({\user2{r}}))=- ({\user2{B}}({\user2{r}})\times {\user2{R}})\times {\user2{e}}), \end{aligned}$$

where the last equality follows from fact that the cross product satisfies the Jacobi identity. Moreover

$$\begin{aligned} ({\user2{e}}\cdot {\user2{R}}){\user2{B}}({\user2{r}})-\big ({\user2{e}}\cdot {\user2{B}}({\user2{r}})\big ){\user2{R}}= \big ({\user2{R}}\times {\user2{B}}({\user2{r}})\big )\times {\user2{e}}. \end{aligned}$$

Finally, applying identity (8) with ${\user2{M}}= {\user2{B}}'({\user2{r}})$ and taking into account that $\nabla \cdot {\user2{B}}({\user2{r}})={\text {Tr}}{\user2{B}'}({\user2{r}}) =0$ we get

$$\begin{aligned} {\user2{B}}^{'T}({\user2{r}})\big ({\user2{S}}({\user2{r}})\times {\user2{e}}\big )-({\user2{B}}'({\user2{r}}){\user2{e}})\times {\user2{S}}({\user2{r}})= -\left( {\user2{B}}'({\user2{r}}){\user2{S}}({\user2{r}})\right) \times {\user2{e}}. \end{aligned}$$

With the above simplification we obtain

90 $$\begin{aligned} \begin{aligned} {\user2{X}}_{F,{\user2{p}}}&= -{\user2{p}}\times {\user2{R}}-d \left[ {\user2{B}}'({\user2{r}}){\user2{S}}({\user2{r}}) + {\user2{B}}({\user2{r}})\times {\user2{R}}\right] \times {\user2{e}}\\&\quad +\frac{d\delta }{2}\Big [B''({\user2{r}})({\user2{e}},{\user2{e}})\times {\user2{S}}({\user2{r}})+ 2\big ({\user2{B}}'({\user2{r}}){\user2{e}}\big )\times ({\user2{R}}\times {\user2{e}})\Big ]\\&=-{\user2{p}}\times {\user2{R}}. \end{aligned} \end{aligned}$$

The last equality occurs because terms in square brackets in the above formula vanish since *F* is by assumption first integral, so conditions (57) and (63b) are fulfilled.

We calculate the other half of components

$$\begin{aligned} \begin{aligned} {\user2{X}}_{F,{\user2{g}}}&=-\varvec{\Omega }^T\frac{\partial F}{\partial {\user2{p}}}-{{\widehat{{\user2{e}}}}} \frac{\partial F}{\partial {\user2{e}}}-\left( {{\widehat{{\user2{g}}}}} +d\delta ({\user2{e}}\cdot {\user2{B}}({\user2{r}})){{\widehat{{\user2{e}}}}}\right) \frac{\partial F}{\partial {\user2{g}}}\\&= -d {\user2{B}}({\user2{r}}) {\user2{e}}^T{\user2{S}}({\user2{r}}) + d({\user2{e}}\cdot {\user2{B}}){\user2{S}}({\user2{r}}) +d{\user2{e}}\times \Big ({\user2{B}}({\user2{r}})\times {\user2{S}}({\user2{r}})\Big )-{\user2{g}}\times {\user2{R}}\\&\quad - d\delta \Big [ \left( {\user2{B}}'({\user2{r}}){\user2{e}}\right) {\user2{e}}^{T} {\user2{S}}({\user2{r}})- \left( {\user2{e}}\cdot \left( {\user2{B}}'({\user2{r}}){\user2{e}}\right) \right) {\user2{S}}({\user2{r}})+ ({\user2{e}}\cdot {\user2{B}}){\user2{e}}\times {\user2{R}}\Big ] \\&=-{\user2{g}}\times {\user2{R}}-d\delta \Big [{\user2{e}}\times \left( ({\user2{B}}'({\user2{r}}){\user2{e}})\times {\user2{S}}({\user2{r}})\right) + ({\user2{e}}\cdot {\user2{B}}({\user2{r}})){\user2{e}}\times {\user2{R}}\Big ]=-{\user2{g}}\times {\user2{R}}, \end{aligned} \end{aligned}$$

the last equality occurs because the term in the square backed vanishes according to condition (63a). $\square$

## Final notes and comments

We showed that equations of motion of a small dipole are Hamiltonian with respect to a certain Poisson structure. It is not trivial as a truncation of Hamilton’s equation does not give Hamiltonian equations in general.

Fact that the existence of a first integral linear in momenta is connected with certain symmetry of the system is well known. For a single charged particle motion in stationary electromagnetic fields the results of group symmetry analysis are known, see, for example^12,13^. However system considered in this paper is much more complicated – it has five degrees of freedom and the form of electromagnetic field is not specified. This is why symmetry analysis of the system can be perform only in restricted cases. We show that if equations of motion for a small dipole admit a first integral linear with respect variables $({\user2{p}}, {\user2{g}}, {\user2{e}})$, then they possess a linear non-homogeneous Hamiltonian symmetry field. The proof of Lemma 4.13 shows that this fact is not obvious. This symmetry condition restricts strongly fields ${\user2{E}}({\user2{r}})$ and ${\user2{B}}({\user2{r}})$ – they have to commute with linear vector field ${\user2{S}}({\user2{r}})={\user2{C}}+ {\user2{R}}\times {\user2{r}}$. Thanks to this we were able to classify electromagnetic fields which admit the prescribed first integral.

As we already mentioned, it is hopeless to investigate integrability of the system without explicit specification of fields ${\user2{E}}({\user2{r}})$ and ${\user2{B}}({\user2{r}})$. As we shown in^4^, even if we assume that these fields are constant the system is not integrable in general. It appears that the relative orientation of these fields is important. As integrability analysis presented in^4^ concerns the reduced form of equations (42) here we give several remarks concerning the integrability of unreduced equations of motion.

For constant ${\user2{E}}$ and ${\user2{B}}$ first integrals linear in ${\user2{p}}$, ${\user2{e}}$ and ${\user2{g}}$ always exit. It is enough to take ${\user2{R}}=\varvec{0}$ and arbitrary ${\user2{C}}$ in formula (55), then all conditions $R_{i,j,k}=0$ with $R_{i,j,k}$ given by (56a)–(56e) are fulfilled. Thus we have three first integrals given by components of

91 $$\begin{aligned} {\user2{Q}}={\user2{p}}-d{\user2{e}}\times {\user2{B}}. \end{aligned}$$

Integrals *H*, $H_0$, $H_1$ and ${\user2{Q}}$ are functionally independent and pairwise commute but for the integrability of the system still one first integral is missing. In our previous work^4^ we used integrals ${\user2{Q}}$ for the reduction of the equations of motion of the dipole to a Hamiltonian system with two degrees of freedom.

If $\delta =0$, then for constant electric and magnetic fields conditions $R_{i,j,k}=0$ with $R_{i,j,k}$ given by (56a)–(56e) reduce to two vector conditions ${\user2{R}}\times {\user2{E}}=\varvec{0}$ and ${\user2{R}}\times {\user2{B}}=\varvec{0}$. In the case when vectors ${\user2{R}}$, ${\user2{B}}$ and ${\user2{E}}$ are parallel, substitution ${\user2{C}}=\varvec{0}$ in formula (55) gives additional first integral

92 $$\begin{aligned} F=({\user2{R}}\times {\user2{r}})\cdot ({\user2{p}}-d {\user2{e}}\times {\user2{B}})+{\user2{R}}\cdot {\user2{g}}=({\user2{R}}\times {\user2{r}})\cdot {\user2{Q}}+{\user2{R}}\cdot {\user2{g}}. \end{aligned}$$

Integrals *H*, $H_0$, $H_1$, components of ${\user2{Q}}$ and *F* are functionally independent but *F* does not commute with ${\user2{Q}}$ because

$$\begin{aligned} \{F,{\user2{Q}}\}={\user2{p}}\times {\user2{R}}+d({\user2{R}}\times {\user2{e}})\times {\user2{B}}. \end{aligned}$$

If ${\user2{R}}\ne 0$ and $\delta \ne 0$, then integral of the form (55) exists only for ${\user2{B}}=\varvec{0}$. But if $\delta \ne 0$ and ${\user2{E}}\times {\user2{B}}=\varvec{0}$ then, except first integral ${\user2{Q}}$, an additional first integral functionally independent of ${\user2{Q}}$ exists. It has the form

93 $$\begin{aligned} T={\user2{p}}\cdot ({\user2{B}}\times {\user2{r}})+d{\user2{B}}\cdot \left( {\user2{e}}\times ({\user2{B}}\times {\user2{r}})\right) + {\user2{B}}\cdot {\user2{g}}-\frac{d\delta }{2}({\user2{B}}\cdot {\user2{e}})^2. \end{aligned}$$

But it also gives non-vanishing Poisson brackets: $\{T,{\user2{Q}}\}={\user2{Q}}\times {\user2{B}}.$

In the case when $\delta =0$ for arbitrary constant fields ${\user2{E}}$ and ${\user2{B}}$ there exists one more functionally independent first integral which is not of the form (55) and it reads

94 $$\begin{aligned} S=({\user2{B}}\cdot {\user2{Q}})\Big ({\user2{B}}\cdot ({\user2{g}}+{\user2{r}}\times {\user2{Q}})\Big )+m {\user2{r}}\cdot \left( {\user2{Q}}\times ({\user2{E}}\times {\user2{B}})\right) . \end{aligned}$$

But this additional first integral does not imply integrability because it does not commute with components of ${\user2{Q}}$, namely we have three non-zero Poisson brackets

$$\begin{aligned} \{S,{\user2{Q}}\}=({\user2{B}}\cdot {\user2{Q}})({\user2{Q}}\times {\user2{B}}) + m( ({\user2{B}}\times {\user2{E}})\times {\user2{Q}}). \end{aligned}$$

In the case when $\delta =0$ and additionally, vectors ${\user2{R}}$, ${\user2{B}}$ and ${\user2{E}}$ are parallel, we have first integrals $H_0,H_1,H,{\user2{Q}}, F,T$ and *S* which are functionally dependent. Among them seven functions are functionally independent but we cannot choose a commutative set of first integrals. Looking on non-vanishing commutators $\{F,{\user2{Q}}\}$, $\{T,{\user2{Q}}\}$ and $\{S,{\user2{Q}}\}$ one can built first integral $K=({\user2{B}}\cdot {\user2{Q}}){\user2{T}}-{\user2{S}}$, such that $\{K,{\user2{Q}}\}=\varvec{0}$ but it is functionally dependent on $H_0,H_1,H,{\user2{Q}}$.

In the case when ${\user2{B}}=\varvec{0}$ and ${\user2{E}}=[E_1,E_2,E_3]^T={\text {const}}$ first integrals ${\user2{Q}}$ simplify just to linear momenta of the centre of mass ${\user2{Q}}={\user2{p}}$, moreover also angular momentum ${\user2{K}}={\user2{r}}\times {\user2{p}}$ and function $G={\user2{E}}\cdot {\user2{g}}$ are preserved during evolution of the system. Among them functionally independent is the set of first integrals $H_0,H_1,H,p_1,p_2,p_3,K_1,K_2,G$. This system is integrable because first integrals $H_0,H_1,H,p_1,p_2,p_3,G$ commute each other. In fact it is maximally super-integrable with two additional first integrals $K_1,K_2$. Non-vanishing Poisson brackets of first integrals are following

95 $$\begin{aligned} \{p_i,K_j\}=\varepsilon _{ijk}p_k, \qquad \{K_i,K_j\}=\varepsilon _{ijk}K_k. \end{aligned}$$

In this case translational motion of the centre of mass separates from rotational motion and the centre of mass moves with constant momentum ${\user2{p}}(t)={\user2{p}}_0$ along straight line ${\user2{r}}(t)=\tfrac{{\user2{p}}_0}{m}t+{\user2{r}}_0.$ Equations of motion for rotational motion simplify to

96 $$\begin{aligned} {{\dot{{\user2{e}}}}} = \frac{1}{I}{\user2{g}}\times {\user2{e}},\quad {{\dot{{\user2{g}}}}} = d{\user2{e}}\times {\user2{E}}. \end{aligned}$$

This system has three obvious first integrals

97 $$\begin{aligned} H=\frac{1}{2I}{\user2{g}}\cdot {\user2{g}}-d {\user2{e}}\cdot {\user2{E}}, \qquad H_0= {\user2{e}}\cdot {\user2{e}}, \qquad H_1= {\user2{e}}\cdot {\user2{g}}. \end{aligned}$$

It has an additional first integral so it is integrable on the common level of geometric integrals $H_0=1$ and $H_1=0$. To see this explicitly let us parametrise vector ${\user2{e}}$ by spherical coordinates

98 $$\begin{aligned} e_1=\cos (q_2)\sin (q_1), \qquad e_2=\sin (q_2)\sin (q_1),\qquad e_3=\cos (q_1). \end{aligned}$$

The canonical momenta conjugated with $(q_1,q_2)$ are $p_1= I \dot{q}_1$ and $p_1= I \sin (q_1)^2 \dot{q}_2$. By a proper choice of the inertial frame we achieve that ${\user2{E}}=(0,0,E)$. In these canonical variables the Hamiltonian function reads

99 $$\begin{aligned} H=\frac{1}{2I} \left( p_1^2 + \frac{p_2^2}{\sin (q_1)^2}\right) - d E \cos (q_1). \end{aligned}$$

Clearly $q_2$ is a cyclic variable so $p_2$ is a first integral. Fixing its value we obtain a system with one degree of freedom. It can be solved explicitly in terms of elliptic function. In fact, the Hamiltonian (99) describes a spherical pendulum for which explicit solutions can be found for example in par. 55 of^14^.

One can continue search of first integrals of system (42) which are polynomial of degree two with respect to variables ${\user2{p}}$, ${\user2{g}}$ and ${\user2{e}}$. Vanishing of time derivative of such first integral gives a non-homogeneous polynomial of degree four of variables $({\user2{p}},{\user2{e}},{\user2{g}})$. Analysis of obtained 22 conditions which are homogeneous polynomials of degree 1, 2, 3 or 4 in these variables has appeared to be much more complicated because system (42) possesses already certain quadratic first integrals: Hamiltonian *H* and Casimir functions $H_0$ and $H_1$ and we were not able to complete it.

Considered equations of motion were obtained in non-relativistic framework under assumption that velocities of material points are very small in comparison with the velocity of light.

We did not consider the energy losses resulting from the fact that a charge moving with acceleration emits electromagnetic waves which carries the energy, the momentum and the angular momentum. The change of energy of an accelerated point charge in time is described by means of famous Larmor’s formula

100 $$\begin{aligned} \frac{\mathrm {d}E}{\mathrm {d}t } =\tau a^2, \quad \tau =\tfrac{\mu _0 q}{6\pi c}, \end{aligned}$$

where $\mu _0$ is the vacuum permeability, *q* is the charge of the particle and ${\user2{a}}$ is its acceleration, see, for example, Sec 11.2.1 in^7^. Since the dipole is created by two charges of opposite signs which radiate independently its energy loss is

101 $$\begin{aligned} \frac{\mathrm {d}E_{\mathrm {dip}}}{\mathrm {d}t }= & {} \tau ({\ddot{{\user2{r}}}}_1^2+{\ddot{{\user2{r}}}}_2^2)\\= & {} \tau \left[ 2{\ddot{{\user2{r}}}}^2+\frac{m_1^2+m_2^2}{m^2}l^2\left( \omega ^4+({{\dot{\varvec{\omega }}}}\times {\user2{e}})^2\right) +2\delta {\ddot{{\user2{r}}}}\times \left( {{\dot{\varvec{\omega }}}}\times {\user2{e}}-\varvec{\omega }^2{\user2{e}}\right) \right] ,\quad \varvec{\omega }={\user2{e}}\times {{\dot{{\user2{e}}}}}=\frac{1}{I}{\user2{g}}. \end{aligned}$$

To neglect the radiation phenomenon of an accelerating dipole, the translational acceleration of its centre of mass and the angular velocity related to the rotation of the dipole around its centre of mass must be sufficiently small.

Loss of the energy, the momentum and the angular momentum means damping of motion of the point charge. Using conservation of energy and Larmor’s formula (100) one can obtain the so-called Abraham–Lorentz formula for the radiation damping force

102 $$\begin{aligned} {\user2{F}}_{\mathrm {rad}}=\frac{\mu _0 q^2}{6\pi c} {{\dot{{\user2{a}}}}} =\frac{2}{3} \frac{q^2}{4\pi \varepsilon _0 c^3} {{\dot{{\user2{a}}}}}, \end{aligned}$$

where $\varepsilon _0$ is the vacuum permittivity, see, for example, Sec. 11.2.2 in^7^, Sec. 75 in^15^ or^16^, which we should add to the right-hand side of the Newton–Lorentz equation (9) if we want to take into account radiation phenomena.

Although that the Abraham–Lorentz formula is commonly accepted non-relativistic expression for the radiation damping force acting on a point charge in an external electromagnetic field it suffers from certain nonphysical solutions: runaway solutions and preacceleration, for more details see, for example, Sec. 11.2.2 in^7^ or^16^. One of the most frequently applied remedies is to use the Landau-Lifshitz formula as an alternative to the Abraham–Lorentz expression. The Landau-Lifshitz formula is obtained by expressing the time derivative of acceleration in (102) by means of the acting external forces (neglecting the radiation damping force), thus by electromagnetic field and its derivatives, for details see Sec. 75 in^15^. These difficulties also persist in the relativistic version of the Abraham-Lorentz formula called the Lorentz-Abraham-Dirac formula and there is a relativistic version of the Landau-Lifshitz formula described for example in Sec. 76 of^15^.

## Appendix 1: Lagrange equations for two opposite sign point charges with a fixed distance between them

The Lagrange function of two point masses $m_1$ and $m_2$ with charges $+q$ and $-q$ localised at ${\user2{r}}_1$ and ${\user2{r}}_2$,respectively, has the form

103 $$\begin{aligned} L=\frac{1}{2}\left( m_1{\dot{{\user2{r}}}}_1^2+m_2{\dot{{\user2{r}}}}_2^2\right) -q\Phi ({\user2{r}}_1)+q\Phi ({\user2{r}}_2) +q\left( {\dot{{\user2{r}}}}_1{\user2{A}}({\user2{r}}_1)\right) - q\left( {\dot{{\user2{r}}}}_2{\user2{A}}({\user2{r}}_2)\right) , \end{aligned}$$

where $\Phi ({\user2{r}})$ and ${\user2{A}}({\user2{r}})$ are scalar and vector potentials of the electromagnetic field defined by the formulae ${\user2{E}}({\user2{r}}) =-\nabla \Phi ({\user2{r}})$ and ${\user2{B}}({\user2{r}}) =\nabla \times {\user2{A}}({\user2{r}})$.

We assume that the distance *l* between charges ${\user2{r}}_1-{\user2{r}}_2=l{\user2{e}}$ is fixed thus we have to take into account the presence of the constrain ${\user2{e}}^2=1$. Moreover, from the above Lagrangian we subtract the total time derivative

104 $$\begin{aligned} \frac{q}{2}\frac{\mathrm {d}}{\mathrm {d}t}\left( (\delta +l){\user2{e}}\cdot {\user2{A}}({\user2{r}}_1) -(\delta -l){\user2{e}}\cdot {\user2{A}}({\user2{r}}_2)\right) . \end{aligned}$$

Thus as result we consider the following Lagrange function

105 $$\begin{aligned} \begin{aligned} {{\mathcal {L}}}&=\frac{1}{2}\left( m_1{\dot{{\user2{r}}}}_1^2+m_2{\dot{{\user2{r}}}}_2^2\right) -q\Phi ({\user2{r}}_1)+q\Phi ({\user2{r}}_2) +q{\dot{{\user2{r}}}}_1\cdot {\user2{A}}({\user2{r}}_1)- q{\dot{{\user2{r}}}}_2\cdot {\user2{A}}({\user2{r}}_2)\\&\quad -\frac{q}{2}\frac{\mathrm {d}}{\mathrm {d}t}\Big [(\delta +l) {\user2{e}}\cdot {\user2{A}}({\user2{r}}_1)-(\delta -l){\user2{e}}\cdot {\user2{A}}({\user2{r}}_2)\Big ]-\lambda ({\user2{e}}^2-1). \end{aligned} \end{aligned}$$

Taking into account definitions of the centre of mass radius vector ${\user2{r}}$ and the dipole vector ${\user2{e}}$ given in (12) expression for ${{\mathcal {L}}}$ can be rewritten as

106 $$\begin{aligned} \begin{aligned} {{\mathcal {L}}}&=\frac{1}{2}m{{\dot{{\user2{r}}}}}^2+\frac{1}{2}I{{\dot{{\user2{e}}}}}^2 -q \Phi ({\user2{r}}_1)+q\Phi ({\user2{r}}_2) +q{{\dot{{\user2{r}}}}}\cdot {\user2{A}}({\user2{r}}_1)-q{{\dot{{\user2{r}}}}}\cdot {\user2{A}}({\user2{r}}_2)\\&\quad -\frac{q}{2}(\delta +l){\user2{e}}\cdot {\user2{A}}'({\user2{r}}_1){{\dot{{\user2{r}}}}}+\frac{q}{2} (\delta -l){\user2{e}}\cdot {\user2{A}}'({\user2{r}}_2){{\dot{{\user2{r}}}}}\\&\quad -\frac{q}{4}(\delta +l)^2{\user2{e}}\cdot {\user2{A}}'({\user2{r}}_1){{\dot{{\user2{e}}}}} +\frac{q}{4}(\delta -l)^2{\user2{e}}\cdot {\user2{A}}'({\user2{r}}_2){{\dot{{\user2{e}}}}}-\lambda ({\user2{e}}^2-1), \end{aligned} \end{aligned}$$

where we also used identity $m_1(\delta +l)+m_2(\delta -l)=0$. To obtain the first Lagrange equation we need to calculate derivatives

$$\begin{aligned} \begin{aligned} \frac{\partial {{\mathcal {L}}}}{\partial {{\dot{{\user2{r}}}}}}&=m{{\dot{{\user2{r}}}}}+q{\user2{A}}({\user2{r}}_1)- q{\user2{A}}({\user2{r}}_2) -\frac{q}{2}(\delta +l){\user2{A}}^{'T}({\user2{r}}_1){\user2{e}}+\frac{q}{2}(\delta -l){\user2{A}}^{'T}({\user2{r}}_2){\user2{e}},\\ \frac{\partial {{\mathcal {L}}}}{\partial {\user2{r}}}&=q{\user2{E}}({\user2{r}}_1)-q{\user2{E}}({\user2{r}}_2)+q{\user2{A}}^{'T}({\user2{r}}_1){{\dot{{\user2{r}}}}}- q{\user2{A}}^{'T}({\user2{r}}_2){{\dot{{\user2{r}}}}}-\frac{q}{2}(\delta +l){\user2{A}}''({\user2{r}}_1)({\user2{e}},{{\dot{{\user2{r}}}}}) +\frac{q}{2}(\delta -l){\user2{A}}''({\user2{r}}_2)({\user2{e}},{{\dot{{\user2{r}}}}})\\&\quad -\frac{q}{4}(\delta +l)^2{\user2{A}}''({\user2{r}}_1)({\user2{e}},{{\dot{{\user2{e}}}}}) +\frac{q}{4}(\delta -l)^2{\user2{A}}''({\user2{r}}_2)({\user2{e}},{{\dot{{\user2{e}}}}}),\\ \frac{\mathrm {d}}{\mathrm {d}t}\left( \frac{\partial {{\mathcal {L}}}}{\partial {{\dot{{\user2{r}}}}}}\right)&= m{\ddot{{\user2{r}}}}+q{\user2{A}}'({\user2{r}}_1){{\dot{{\user2{r}}}}}-q{\user2{A}}'({\user2{r}}_2){{\dot{{\user2{r}}}}} +\frac{q}{2}(\delta +l){\user2{A}}'({\user2{r}}_1){{\dot{{\user2{e}}}}} -\frac{q}{2}(\delta -l){\user2{A}}'({\user2{r}}_2){{\dot{{\user2{e}}}}}\\&\quad -\frac{q}{2}(\delta +l){\user2{A}}''({\user2{r}}_1)({\user2{e}},{{\dot{{\user2{r}}}}}) +\frac{q}{2}(\delta -l){\user2{A}}''({\user2{r}}_2)({\user2{e}},{{\dot{{\user2{r}}}}})\\&\quad -\frac{q}{4}(\delta +l)^2{\user2{A}}''({\user2{r}}_1)({\user2{e}},{{\dot{{\user2{e}}}}}) +\frac{q}{4}(\delta -l)^2{\user2{A}}''({\user2{r}}_2)({\user2{e}},{{\dot{{\user2{e}}}}}) -\frac{q}{2}(\delta +l){\user2{A}}^{'T}({\user2{r}}_1){{\dot{{\user2{e}}}}} +\frac{q}{2}(\delta -l){\user2{A}}^{'T}({\user2{r}}_2){{\dot{{\user2{e}}}}}. \end{aligned} \end{aligned}$$

As result we obtain

107 $$\begin{aligned} \frac{\mathrm {d}}{\mathrm {d}t}\left( \frac{\partial {{\mathcal {L}}}}{\partial {{\dot{{\user2{r}}}}}}\right) -\frac{\partial {{\mathcal {L}}}}{\partial {\user2{r}}}= & {} m{\ddot{{\user2{r}}}}-q[{\user2{e}}({\user2{r}}_1)-{\user2{E}}({\user2{r}}_2)]-q{{\dot{{\user2{r}}}}} \times \left[ {\user2{B}}({\user2{r}}_1)-{\user2{B}}({\user2{r}}_2)\right] \\&-\frac{q}{2}{{\dot{{\user2{e}}}}}\times \left[ (\delta +l) {\user2{B}}({\user2{r}}_1)-(\delta -l){\user2{B}}({\user2{r}}_2)\right] =0. \end{aligned}$$

We used $\Phi '({\user2{r}}_i )=-{\user2{E}}({\user2{r}}_i)$ and identities

108 $$\begin{aligned} {\user2{A}}'({\user2{r}}_i){{\dot{{\user2{r}}}}}- {\user2{A}}^{'T}({\user2{r}}_i){{\dot{{\user2{r}}}}} ={\user2{B}}({\user2{r}}_i)\times {{\dot{{\user2{r}}}}},\qquad {\user2{A}}'({\user2{r}}_i){{\dot{{\user2{e}}}}}- {\user2{A}}^{'T}({\user2{r}}_i){{\dot{{\user2{e}}}}} ={\user2{B}}({\user2{r}}_i)\times {{\dot{{\user2{e}}}}}, \end{aligned}$$

for $$i=1,2$$.

Now we calculate derivatives of ${{\mathcal {L}}}$ with respect to ${{\dot{{\user2{e}}}}}$ and ${\user2{e}}$

$$\begin{aligned} \begin{aligned} \frac{\partial {{\mathcal {L}}}}{\partial {{\dot{{\user2{e}}}}}}&= I{{\dot{{\user2{e}}}}}-\frac{q}{4}(\delta +l)^2{\user2{A}}^{'T}({\user2{r}}_1){\user2{e}}+\frac{q}{4}(\delta -l)^2{\user2{A}}^{'T}({\user2{r}}_2){\user2{e}},\\ \frac{\partial {{\mathcal {L}}}}{\partial {\user2{e}}}&= \frac{q}{2}(\delta +l){\user2{E}}({\user2{r}}_1) -\frac{q}{2}(\delta -l){\user2{E}}({\user2{r}}_2)+\frac{q}{2}(\delta +l){\user2{A}}^{'T}({\user2{r}}_1){{\dot{{\user2{r}}}}} -\frac{q}{2}(\delta -l){\user2{A}}^{'T}({\user2{r}}_2){{\dot{{\user2{r}}}}} -\frac{q}{2}(\delta +l){\user2{A}}'({\user2{r}}_1){{\dot{{\user2{r}}}}}\\&\quad +\frac{q}{2}(\delta -l){\user2{A}}'({\user2{r}}_2){{\dot{{\user2{r}}}}} -\frac{q}{4}(\delta +l)^2{\user2{A}}''({\user2{r}}_1)\left( {\user2{e}},{{\dot{{\user2{r}}}}}\right) +\frac{q}{4}(\delta -l)^2{\user2{A}}''({\user2{r}}_2)\left( {\user2{e}},{{\dot{{\user2{r}}}}}\right) -\frac{q}{4}(\delta +l)^2{\user2{A}}'({\user2{r}}_1){{\dot{{\user2{e}}}}}\\&\quad +\frac{q}{4}(\delta -l)^2{\user2{A}}'({\user2{r}}_2){{\dot{{\user2{e}}}}} -\frac{q}{8}(\delta +l)^3{\user2{A}}''({\user2{r}}_1)\left( {\user2{e}},{{\dot{{\user2{e}}}}}\right) + \frac{q}{8}(\delta -l)^3{\user2{A}}''({\user2{r}}_2)\left( {\user2{e}},{{\dot{{\user2{e}}}}}\right) +2\lambda {\user2{e}},\\ \frac{\mathrm {d}}{\mathrm {d}t}\left( \frac{\partial {{\mathcal {L}}}}{\partial {{\dot{{\user2{e}}}}}}\right)&=I{\ddot{{\user2{e}}}}-\frac{q}{4}(\delta +l)^2{\user2{A}}''({\user2{r}}_1)\left( {\user2{e}},{{\dot{{\user2{r}}}}}\right) +\frac{q}{4}(\delta -l)^2{\user2{A}}''({\user2{r}}_2)\left( {\user2{e}},{{\dot{{\user2{r}}}}}\right) -\frac{q}{8}(\delta +l)^3{\user2{A}}''({\user2{r}}_1)\left( {\user2{e}},{{\dot{{\user2{e}}}}}\right) \\&\quad +\frac{q}{8}(\delta -l)^3{\user2{A}}''({\user2{r}}_2)\left( {\user2{e}},{{\dot{{\user2{e}}}}}\right) -\frac{q}{4}(\delta +l)^2{\user2{A}}^{'T}({\user2{r}}_1){{\dot{{\user2{e}}}}} +\frac{q}{4}(\delta -l)^2{\user2{A}}^{'T}({\user2{r}}_2){{\dot{{\user2{e}}}}}. \end{aligned} \end{aligned}$$

The second Lagrange equation takes the form

109 $$\begin{aligned} \begin{aligned} \frac{\mathrm {d}}{\mathrm {d}t}\left( \frac{\partial {{\mathcal {L}}}}{\partial {{\dot{{\user2{e}}}}}}\right) -\frac{\partial {{\mathcal {L}}}}{\partial {\user2{e}}}&= I{\ddot{{\user2{e}}}}-\frac{q}{2}\left[ (\delta +l){\user2{E}}({\user2{r}}_1)-(\delta -l){\user2{E}}({\user2{r}}_2)\right] \\&\quad -\frac{q}{2}{{\dot{{\user2{r}}}}}\times \Big [(\delta +l){\user2{B}}({\user2{r}}_1)-(\delta -l){\user2{B}}({\user2{r}}_2)\Big ]\\&\quad -\frac{q}{4}{{\dot{{\user2{e}}}}}\times \Big [(\delta +l)^2{\user2{B}}({\user2{r}}_1)-(\delta -l)^2{\user2{B}}({\user2{r}}_2)\Big ]+2\lambda {\user2{e}}=0. \end{aligned} \end{aligned}$$

When we apply the cross product of ${\user2{e}}$ from the left to this equation and we recall that ${\user2{e}}\times I{\ddot{{\user2{e}}}}=I{{\dot{\varvec{\omega }}}}={{\dot{{\user2{g}}}}}$, then (109) can be rewritten as

110 $$\begin{aligned} \begin{aligned} {{\dot{{\user2{g}}}}}&= \frac{q}{2}{\user2{e}}\times \left[ (\delta +l){\user2{E}}({\user2{r}}_1) -(\delta -l){\user2{E}}({\user2{r}}_2)\right] +\frac{q}{2}{\user2{e}}\times \Big [{{\dot{{\user2{r}}}}} \times \Big ((\delta +l){\user2{B}}({\user2{r}}_1)-(\delta -l){\user2{B}}({\user2{r}}_2)\Big )\Big ]\\&\quad +\frac{q}{4}\Big [(\delta +l)^2{\user2{e}}\cdot {\user2{B}}({\user2{r}}_1)-(\delta -l)^2 {\user2{e}}\cdot {\user2{B}}({\user2{r}}_2)\Big ]{{\dot{{\user2{e}}}}}, \end{aligned} \end{aligned}$$

where we used the well known formula for the vector triple product. When we recall definitions of linear and angular momenta, then we immediately obtain system (23) from the above Lagrange equations (107) and (110).

## Appendix 2: Lagrange equations for a small dipole

The length of dipole *l* is small, so it is justifiable to expand the scalar and vector potentials into power series with respect to *l* up to a certain order. In further considerations it is necessary to keep terms up to the second order. Expansions of the potentials have the following forms

111 $$\begin{aligned} \begin{aligned} \Phi ({\user2{r}}_1)&= \Phi ({\user2{r}})+\frac{1}{2}(\delta +l)\Phi '({\user2{r}})\cdot {\user2{e}}+ \frac{1}{8}(\delta +l)^2{\user2{e}}\cdot \left( \Phi ''({\user2{r}}){\user2{e}}\right) ,\\ \Phi ({\user2{r}}_2)&= \Phi ({\user2{r}})+\frac{1}{2}(\delta -l)\Phi '({\user2{r}})\cdot {\user2{e}}+ \frac{1}{8}(\delta -l)^2{\user2{e}}\cdot \left( \Phi ''({\user2{r}}){\user2{e}}\right) ,\\ {\user2{A}}({\user2{r}}_1)&={\user2{A}}({\user2{r}})+\frac{1}{2}(\delta +l){\user2{A}}'({\user2{r}}){\user2{e}}+\frac{1}{8}(\delta +l)^2{\user2{A}}''({\user2{r}})({\user2{e}},{\user2{e}}),\\ {\user2{A}}({\user2{r}}_2)&={\user2{A}}({\user2{r}})+\frac{1}{2}(\delta -l){\user2{A}}'({\user2{r}}){\user2{e}}+\frac{1}{8}(\delta -l)^2{\user2{A}}''({\user2{r}})({\user2{e}},{\user2{e}}). \end{aligned} \end{aligned}$$

Let us notice that if we expand the total time derivative given in (104) to the first order terms, then we obtain

$$\begin{aligned} \begin{aligned}{}&\frac{q}{2}\frac{\mathrm {d}}{\mathrm {d}t}\left[ (\delta +l)\user2{e}\cdot \user2{A}(\user2{r}_1)- (\delta -l)\user2{e}\cdot \user2{A}(\user2{r}_2)\right] \\&\quad =\frac{q}{2}\frac{\mathrm {d}}{\mathrm {d}t} \Big [(\delta +l)\user2{e}\cdot \user2{A}(\user2{r}) +\frac{1}{2}(\delta +l)^2\user2{e}\cdot \left( \user2{A}'(\user2{r})\user2{e}\right) - (\delta -l)\user2{e}\cdot \user2{A}(\user2{r})-\frac{1}{2}(\delta -l)^2\user2{e}\cdot \left( \user2{A}'(\user2{r})\user2{e}\right) \Big ]+\cdots \\&\quad =q\frac{\mathrm {d}}{\mathrm {d}t}\left[ l\user2{e}\cdot \user2{A}(\user2{r})+\frac{1}{2}\delta l\user2{e}\cdot \left( \user2{A}'(\user2{r})\user2{e}\right) \right] +\cdots . \end{aligned} \end{aligned}$$

When we substitute expansions (111) into (103) and subtract terms which are the total time derivatives of $d{\user2{e}}\cdot {\user2{A}}({\user2{r}})$ and of $\frac{1}{2}d\delta {\user2{e}}\cdot ({\user2{A}}'({\user2{r}}){\user2{e}})$, that they do not change the equations of motion, then the truncated Lagrangian has the form

112 $$\begin{aligned} \begin{aligned} L&=\frac{1}{2}m{{\dot{{\user2{r}}}}}^2 + \frac{1}{2}I{{\dot{{\user2{e}}}}}^2 +d\left( -\Phi '({\user2{r}})\cdot {\user2{e}}+{{\dot{{\user2{r}}}}}\cdot {\user2{A}}'({\user2{r}}){\user2{e}}+{{\dot{{\user2{e}}}}}\cdot {\user2{A}}\right) \\&\quad +\frac{d\delta }{2}\left( -{\user2{e}}\cdot \left( \Phi ''({\user2{r}}){\user2{e}}\right) +{{\dot{{\user2{r}}}}}\cdot \left( {\user2{A}}''({\user2{r}})({\user2{e}},{\user2{e}}) \right) +2{{\dot{{\user2{e}}}}}\cdot {\user2{A}}'({\user2{r}}){\user2{e}}\right) \\&\quad -d\left( {{\dot{{\user2{e}}}}}\cdot {\user2{A}}({\user2{r}})+{\user2{e}}\cdot {\user2{A}}'({\user2{r}}){{\dot{{\user2{r}}}}}\right) -\frac{d\delta }{2}\left( {{\dot{{\user2{e}}}}}\cdot {\user2{A}}'({\user2{r}}){\user2{e}}+{\user2{e}}\cdot \left( {\user2{A}}''({\user2{r}})({{\dot{{\user2{r}}}}},{\user2{e}})\right) +{\user2{e}}\cdot {\user2{A}}'({\user2{r}}){{\dot{{\user2{e}}}}}\right) \\&=\frac{1}{2}m{{\dot{{\user2{r}}}}}^2 + \frac{1}{2}I{{\dot{{\user2{e}}}}}^2 +d{\user2{e}}\cdot {\user2{E}}({\user2{r}}) - d{{\dot{{\user2{r}}}}}\cdot \left( {\user2{e}}\times {\user2{B}}({\user2{r}})\right) \\&\quad +\frac{1}{2}d\delta \left[ {\user2{e}}\cdot ({\user2{E}}'({\user2{r}}){\user2{e}}) - {{\dot{{\user2{r}}}}}\cdot ({\user2{e}}\times ({\user2{B}}'({\user2{r}}){\user2{e}})) -{{\dot{{\user2{e}}}}}\cdot ({\user2{e}}\times {\user2{B}}({\user2{r}}))\right] . \end{aligned} \end{aligned}$$

In calculations we used the following identities

$$\begin{aligned} \begin{aligned} {{\dot{{\user2{r}}}}}\cdot {\user2{A}}'({\user2{r}}){\user2{e}}- {\user2{e}}\cdot {\user2{A}}'({\user2{r}}){{\dot{{\user2{r}}}}}&= -{{\dot{{\user2{r}}}}}\cdot ({\user2{e}}\times {\user2{B}}({\user2{r}})),\\ {{\dot{{\user2{e}}}}}\cdot {\user2{A}}'({\user2{r}}){\user2{e}}- {\user2{e}}\cdot {\user2{A}}'({\user2{r}}){{\dot{{\user2{e}}}}}&= -{{\dot{{\user2{e}}}}}\cdot ({\user2{e}}\times {\user2{B}}({\user2{r}})),\\ {{\dot{{\user2{r}}}}}\cdot \left( {\user2{A}}''({\user2{r}})({\user2{e}},{\user2{e}})\right) - {\user2{e}}\cdot \left( {\user2{A}}''({\user2{r}})({{\dot{{\user2{r}}}}},{\user2{e}})\right)&=-{{\dot{{\user2{r}}}}}\left( {\user2{e}}\times ({\user2{B}}'({\user2{r}}){\user2{e}})\right) . \end{aligned} \end{aligned}$$

If we want to treat ${\user2{r}}$ and ${\user2{e}}$ as generalised variables, then we need to take into account constraint ${\user2{e}}^2=1$. In this aim we consider Lagrangian ${{\mathcal {L}}}=L-\lambda ({\user2{e}}^2-1)$. Let us calculate derivatives

$$\begin{aligned} \begin{aligned} \frac{\partial {{\mathcal {L}}}}{\partial {{\dot{{\user2{r}}}}}}&=m{{\dot{{\user2{r}}}}}-d({\user2{e}}\times {\user2{B}})-\frac{d\delta }{2}\left( {\user2{e}}\times {\user2{B}}'({\user2{r}}){\user2{e}}\right) ,\\ \frac{\mathrm {d}}{\mathrm {d}t}\left( \frac{\partial {{\mathcal {L}}}}{\partial {{\dot{{\user2{r}}}}}}\right)&=m{\ddot{{\user2{r}}}}-d\left( {{\dot{{\user2{e}}}}}\times {\user2{B}}+{\user2{e}}\times {\user2{B}}'({\user2{r}}){{\dot{{\user2{r}}}}}\right) -\frac{d\delta }{2}\Big [ {{\dot{{\user2{e}}}}}\times {\user2{B}}'({\user2{r}}){\user2{e}}+{\user2{e}}\times {\user2{B}}''({\user2{r}})({{\dot{{\user2{r}}}}},{\user2{e}}) +{\user2{e}}\times {\user2{B}}'({\user2{r}}){{\dot{{\user2{e}}}}} \Big ],\\ \frac{\partial {{\mathcal {L}}}}{\partial {\user2{r}}}&=d\left( {\user2{E}}'({\user2{r}})^T{\user2{e}}-{\user2{B}}'({\user2{r}})^T ({{\dot{{\user2{r}}}}}\times {\user2{e}})\right) +\frac{d\delta }{2}\left[ {\user2{E}}''({\user2{r}})({\user2{e}},{\user2{e}})-{\user2{B}}''({\user2{r}})\left( {\user2{e}},{{\dot{{\user2{r}}}}}\times {\user2{e}}\right) -{\user2{B}}'({\user2{r}})^T({{\dot{{\user2{e}}}}}\times {\user2{e}})\right] \end{aligned} \end{aligned}$$

and the first Lagrange equation is

113 $$\begin{aligned} \begin{aligned} \frac{\mathrm {d}}{\mathrm {d}t}\left( \frac{\partial {{\mathcal {L}}}}{\partial {{\dot{{\user2{r}}}}}}\right) - \frac{\partial {{\mathcal {L}}}}{\partial {\user2{r}}}&=m{\ddot{{\user2{r}}}} -d\left( {{\dot{{\user2{e}}}}}\times {\user2{B}}+{\user2{e}}\times {\user2{B}}'({\user2{r}}){{\dot{{\user2{r}}}}} +{\user2{E}}'({\user2{r}})^T{\user2{e}}-{\user2{B}}'({\user2{r}})^T({{\dot{{\user2{r}}}}}\times {\user2{e}})\right) \\&\quad -\frac{d\delta }{2}\Big [{{\dot{{\user2{e}}}}}\times {\user2{B}}'({\user2{r}}){\user2{e}}+{\user2{e}}\times {\user2{B}}''({\user2{r}})({{\dot{{\user2{r}}}}},{\user2{e}})+{\user2{e}}\times {\user2{B}}'({\user2{r}}){{\dot{{\user2{e}}}}} +{\user2{E}}''({\user2{r}})({\user2{e}},{\user2{e}})\\&\quad -{\user2{B}}''({\user2{r}})\left( {\user2{e}},{{\dot{{\user2{r}}}}}\times {\user2{e}}\right) -{\user2{B}}'({\user2{r}})^T({{\dot{{\user2{e}}}}}\times {\user2{e}}) \Big ]\\&=m{\ddot{{\user2{r}}}}-d\left( {{\dot{{\user2{e}}}}}\times {\user2{B}}+{\user2{E}}'({\user2{r}})^T{\user2{e}}+{{\dot{{\user2{r}}}}}\times {\user2{B}}'({\user2{r}}){\user2{e}}\right) \\&\quad -\dfrac{d\delta }{2}\left[ {\user2{E}}''({\user2{r}})({\user2{e}},{\user2{e}})+{{\dot{{\user2{r}}}}}\times {\user2{B}}''({\user2{r}})\left( {\user2{e}},{\user2{e}}\right) +2{{\dot{{\user2{e}}}}}\times {\user2{B}}'({\user2{r}}){\user2{e}}\right] =0. \end{aligned} \end{aligned}$$

Let us notice that this equation agrees with the Newton equation of the translational motion of the centre of mass (39) because ${\user2{E}}'({\user2{r}})^T={\user2{E}}'({\user2{r}})$.

In our calculations in equation (113) we used the following identities

$$\begin{aligned} \begin{aligned}{}&{\user2{e}}\times {\user2{B}}'({\user2{r}}){{\dot{{\user2{r}}}}}-{\user2{B}}'({\user2{r}})^T({{\dot{{\user2{r}}}}}\times {\user2{e}}) = {{\dot{{\user2{r}}}}}\times {\user2{B}}'({\user2{r}}){\user2{e}},\\&{{\dot{{\user2{e}}}}}\times {\user2{B}}'({\user2{r}}){\user2{e}}+{\user2{e}}\times {\user2{B}}'({\user2{r}}){{\dot{{\user2{e}}}}} -{\user2{B}}'({\user2{r}})^T({{\dot{{\user2{e}}}}}\times {\user2{e}})=2{{\dot{{\user2{e}}}}}\times {\user2{B}}'({\user2{r}}){\user2{e}},\\&{\user2{e}}\times {\user2{B}}''({\user2{r}})({{\dot{{\user2{r}}}}},{\user2{e}})-{\user2{B}}''({\user2{r}})\left( {\user2{e}},{{\dot{{\user2{r}}}}} \times {\user2{e}}\right) ={{\dot{{\user2{r}}}}}\times {\user2{B}}''({\user2{r}})\left( {\user2{e}},{\user2{e}}\right) , \end{aligned} \end{aligned}$$

which are valid provided $\nabla \cdot {\user2{B}}({\user2{r}}) =0$. To check the last identity is necessary to use vanishing of partial derivatives of the divergence zero condition with respect to all centre of mass variables.

Now we calculate derivatives of ${{\mathcal {L}}}$ with respect to ${\user2{e}}$ and ${{\dot{{\user2{e}}}}}$

$$ \begin{aligned}&\frac{\partial {{\mathcal {L}}}}{\partial {{\dot{\user2{e}}}}}= I{{\dot{\user2{e}}}}-\frac{d\delta }{2}\user2{e}\times \user2{B},\\&\frac{\mathrm {d}}{\mathrm {d}t}\left( \frac{\partial {{\mathcal {L}}}}{\partial {{\dot{\user2{e}}}}}\right) = I{\ddot{\user2{e}}}-\frac{d\delta }{2}\left( {{\dot{\user2{e}}}}\times \user2{B}+\user2{e}\times \user2{B}'(\user2{r}){{\dot{\user2{r}}}}\right) ,\\&\frac{\partial {{\mathcal {L}}}}{\partial \user2{e}}=d\left( \user2{E}-\user2{B}\times {{\dot{\user2{r}}}}\right) -\frac{d\delta }{2} \left[ -2\user2{E}'(\user2{r})\user2{e}+\user2{B}'(\user2{r})\user2{e}\times {{\dot{\user2{r}}}}+\user2{B}'(\user2{r})^T({{\dot{\user2{r}}}}\times \user2{e})+\user2{B}\times {{\dot{\user2{e}}}}\right] -\lambda \user2{e}. \end{aligned}$$

The second Lagrange equation takes the form

114 $$\begin{aligned} \begin{aligned} \frac{\mathrm {d}}{\mathrm {d}t}\left( \frac{\partial {{\mathcal {L}}}}{\partial {{\dot{{\user2{e}}}}}}\right) - \frac{\partial {{\mathcal {L}}}}{\partial {\user2{e}}}&=I{\ddot{{\user2{e}}}}-d\left( {\user2{E}}-{\user2{B}}\times {{\dot{{\user2{r}}}}}\right) \\&\quad - \frac{d\delta }{2}\Big [{{\dot{{\user2{e}}}}}\times {\user2{B}}+ {\user2{e}}\times {\user2{B}}'({\user2{r}}){{\dot{{\user2{r}}}}}+2{\user2{E}}'({\user2{r}}){\user2{e}}-{\user2{B}}'({\user2{r}}){\user2{e}}\times {{\dot{{\user2{r}}}}}-{\user2{B}}'({\user2{r}})^T({{\dot{{\user2{r}}}}}\times {\user2{e}}) -{\user2{B}}\times {{\dot{{\user2{e}}}}}\Big ]+\lambda {\user2{e}}\\&= I{\ddot{{\user2{e}}}}-d\left( {\user2{E}}-{\user2{B}}\times {{\dot{{\user2{r}}}}}\right) -d\delta \left[ {{\dot{{\user2{e}}}}}\times {\user2{B}}+{\user2{E}}'({\user2{r}}){\user2{e}}+{{\dot{{\user2{r}}}}}\times {\user2{B}}'({\user2{r}}){\user2{e}}\right] +\lambda {\user2{e}}=0. \end{aligned} \end{aligned}$$

In our calculations we used the following identity

$$\begin{aligned} {\user2{e}}\times {\user2{B}}'({\user2{r}}){{\dot{{\user2{r}}}}}-{\user2{B}}'({\user2{r}}){\user2{e}}\times {{\dot{{\user2{r}}}}} -{\user2{B}}'({\user2{r}})^T({{\dot{{\user2{r}}}}}\times {\user2{e}})=2{{\dot{{\user2{r}}}}}\times {\user2{B}}'({\user2{r}}){\user2{e}}, \end{aligned}$$

that holds due to vanishing of divergence of ${\user2{B}}$.

Let us recall that $\varvec{\omega }={\user2{e}}\times {{\dot{{\user2{e}}}}}$ and the time derivative ${{\dot{\varvec{\omega }}}}={\user2{e}}\times {\ddot{{\user2{e}}}}$, thus if we take the cross product of ${\user2{e}}$ with the second Lagrange equation (114), then we obtain

115 $$\begin{aligned} I{{\dot{\varvec{\omega }}}} -d\left[ {\user2{e}}\times {\user2{E}}-{\user2{e}}\times ({\user2{B}}\times {{\dot{{\user2{r}}}}})\right] -d\delta \Big [{\user2{e}}\times ({{\dot{{\user2{e}}}}}\times {\user2{B}})+{\user2{e}}\times {\user2{E}}'({\user2{r}}){\user2{e}}+{\user2{e}}\times \left( {{\dot{{\user2{r}}}}}\times {\user2{B}}'({\user2{r}}){\user2{e}}\right) \Big ] =0. \end{aligned}$$

This equation coincides with the Newton equation for the rotational motion given in (41).
